# Hemoglobin level significantly impacts the tumor cell survival fraction in humans after internal radiotherapy

**DOI:** 10.1186/2191-219X-2-20

**Published:** 2012-05-19

**Authors:** Stephan Walrand, Renaud Lhommel, Pierre Goffette, Marc Van den Eynde, Stanislas Pauwels, FranÇois Jamar

**Affiliations:** 1 Department of Nuclear Medicine, Cliniques Universitaires Saint Luc, Universitegrave Catholique de Louvain, Avenue Hippocrate 10, Brussels, 1200, Belgium; 2 Department of Interventional Radiology, Cliniques Universitaires Saint Luc, Universitegrave Catholique de Louvain, Avenue Hippocrate 10, Brussels, 1200, Belgium; 3 Department of Oncology, Cliniques Universitaires Saint Luc, Universitegrave Catholique de Louvain, Avenue Hippocrate 10, Brussels, 1200, Belgium

**Keywords:** Liver SIRT, Internal radiotherapy, Dosimetry, Hemoglobin, Anemia, Dose-response.

## Abstract

**Background:**

Anemia is usually not taken into account in internal radiotherapy. We investigated whether the hemoglobin (Hb) level could have an impact on the tumor response, as observed in external beam radiotherapy (EBRT).

**Methods:**

Absorbed doses of 25 hepatic metastatic sites in eight patients who underwent a liver selective internal radiotherapy (SIRT) were computed by a 3D convolution of a dose deposition kernel with the ^90^Y time-of-flight positron emission tomography (TOF-PET) images acquired following therapy. Early tumor response was assessed by comparing a follow-up FDG TOF-PET scan with a baseline scan. Hb level was measured on the day of the SIRT procedure.

**Results:**

All patients displayed early tumor response increasing with the tumor-absorbed dose. Significant differences between patients were noted, the response slope correlating with the Hb level. After applying a global fit on all metastases using a tumor radiosensitivity modulated by a Hb enhancement factor (HEF) linearly dependent on the Hb level, a strong correlation (*R* = 0.96) was observed between the early response and the absorbed dose. Hb level had a major impact on tumor response by modulating HEF by a factor 6.

**Conclusions:**

These results prove the significant impact of Hb level on the tumor response and support the study of methods for correcting tumor hypoxia, such as intensively performed in EBRT. The quantitative analysis of the relationship between tumor doses and early response has the power to allow fast screening of such correction methods in limited patient series. Internal radiotherapy could be more efficient if performed earlier in the therapy line, when the disease- and treatment-related anemia remains limited.

## Background

The importance of anemia as a prognostic factor of patient outcome in external beam radiotherapy (EBRT) of solid tumors has been documented in large clinical series and thoroughly reviewed [1-8]. This impact results from three related key points: (1) the hemoglobin (Hb) level was shown to strongly correlate with tumor oxygenation in numerous cancers [9], (2) substantial data show that tumor hypoxia is involved in processes conferring a growth advantage and the development of a more malignant phenotype [10-15], and (3) hypoxic tumors are reported to be less sensitive to ionizing radiation since the production of free radicals, which are responsible for lethal DNA breaks, increases with the O_2_ partial pressure (pO_2_) [16-18].

Internal radiotherapy differs from EBRT by higher tumor-absorbed doses delivered at a lower dose rate and with a more heterogeneous pattern. To the best of our knowledge, the impact of hypoxia on tumor response in patients treated by internal radiotherapy has never been directly addressed. Only the effect of Hb level on global symptoms, i.e., pain or cumulative survival, was reported in the treatment for painful osseous metastases in prostate cancer with ^186^Re-hydroxyethylidene-diphosphonate and ^89^Sr [19,20]. The demonstration of a significant impact of anemia on tumor response in internal radiotherapy should allow improvements by considering strategies under intensive development in EBRT [21-26] but, as of today, totally ignored in internal radiotherapy.

Selective internal radiation therapy (SIRT) using ^90^Y-labeled microspheres is a rapidly emerging treatment of unresectable chemorefractory primary liver tumors and hepatic metastases mainly originating from colorectal cancer (CRC). Development of ^90^Y imaging by PET [27] after the SIRT procedure, and recently by pinhole bremsstrahlung SPECT [28], proved that tumor dosimetry is feasible [29-31] and evidenced a promising relationship between absorbed dose and early metabolic response [32] as already observed using ^99m^Tc-MAA-based dosimetry [33]. The aim of this study is to further analyze this relationship and to investigate whether the Hb level measured on the day of the SIRT procedure has an impact on the early tumor response. For this purpose, an estimate of the tumor cell survival fraction was fitted using a tumor radiosensitivity modulated by a hemoglobin enhancement factor (HEF). This radiosensitivity modulation was implemented in the similar way that was performed for the tissue oxygenation when this parameter can be directly assessed [16-18].

## Methods

### Patient series

Eight patients with fludeoxyglucose (FDG)-positive hepatic metastases (six from CRC and two from melanomas) underwent a 45-min ^90^Y time-of-flight positron emission tomography (^90^Y TOF-PET) scan (Gemini TF, Philips Medical Systems, Cleveland, OH, USA) within 4 h following the SIRT procedure. SIRT was performed according to the standard recommendations [34] (mean activity   SD = 1.45   0.45 GBq). Two ^18^ F]FDG PET scans were performed: a baseline (FDG-BL) scan 2.4   2.1 weeks (mean   SD) before SIRT and a follow-up (FDG-FU) scan 6.8   0.8 weeks (mean   SD) after SIRT (see Table 1). All scans were reconstructed using the line of response reconstruction algorithm from Philips Medical Systems (4 iterations × 10 subsets). In all patients, a blood analysis including the Hb level was performed on the day of SIRT. All these procedures are part of the standard therapy as routinely performed in our institution. After approval by the local ethics committee, the metabolic change of 25 tumors before and after therapy was retrospectively analyzed.

**Table 1:** Patients' dosimetric data

**Patient**	**Type**	**Site number**	**Hb (g/dL)**	**BL time (weeks)**	**FU time (weeks)**	**MR**	***D *(Gy)**	**Volume BL (ml)**
1	mel.	1	10.0	−0.2	6.1	0.74	233.2 52.1	11
		2				1.14	130.0 42.3	31
2	CRC	1	13.4	−6.2	8.1	0.11	169.9 79.9	58
		2				4.00	6.1 8.2	17
3	CRC	1	11.1	−3.2	7.2	0.18	172.2 55.8	127
4	CRC	1	14.8	−2.3	6.2	0.15	110.9 33.1	93
		2				6*.*25	52.1 18.5	34
		3				19.52	5.0 10.8	11
		4				20.20	6.4 12.4	25
		5				1.67	18.5 17.0	32
		6				1.43	8.4 11.6	37
		7				3.33	7.9 7.6	6
		8				0.10	131.0 29.8	10
5	CRC	1	14.3	−3.5	7.2	0.05	178.5 35.2	34
		2				1.75	20.4 6.1	6
6	CRC	1	13.0	−2.9	6.1	0.32	113.9 21.9	3
		2				0.34	114.7 14.8	3
		3				0.22	127.3 24.8	4
		4				49.32	14.6 2.8	5
		5				0.86	52.0 9.6	3
		6				0.89	55.5 10.7	4
7	CRC	1	13.1	−0.6	6.1	1.32	52.2 10.6	37
		2				2.08	26.6 6.2	36
8	mel.	1	13.4	−0.2	7.0	0.26	115.0 17.8	7
		2				0.17	147.0 21.2	6

BL, baseline FDG; CRC, colorectal cancer; *D*, dose; FU, follow-up FDG; Hb, hemoglobin; mel., melanoma; MR, tumor metabolic ratio.

### Delineation of volumes of interests

**Figure 1:**
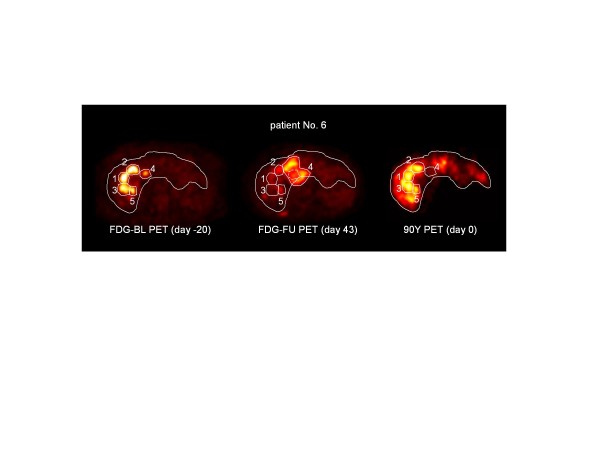
**PET scans.** One transverse slice of the patient No. 6 showing the metabolic sites of its large necrotic tumour that received different absorbed dose. The FDG uptake of the metabolic sites 1,2,3,5, that were well targeted by the microspheres, decreased between the FDG-BL and FDG-FU scans, while the metabolic site 4 not targeted by the microspheres rapidly increased in FDG uptake and in size as well, to become a new large necrotic metastatic site. The VOI on the FDG-FU scan was increased to encompass all the FDG activity originating from the increased site 4.

A total of 25 metastatic sites were identified on the FDG-BL scan (number of sites per patient ranging from one to eight, mean = 3.1). Since the purpose was to study the early response to the absorbed dose (*D*), viable areas of large, partly necrotic tumors were delineated using the FDG-BL scan and were analyzed as individual sites with their own absorbed dose, while the necrotic area was not incorporated in the analysis. Volumes of interest (VOIs) were manually drawn on the fused FDG-BL and ^90^Y-PET images. The same VOIs were used for the evaluation of the FDG-FU scan. Some undertreated viable metastatic sites, i.e., visible on the FDG-BL scan but not well targeted by the microspheres, disclosed a large size increase between the FDG-BL and FDG-FU scans (see Figure 1). In this case, the size of the VOI was then tuned accordingly in order to encompass all the metabolic cells of the metastatic site that were visible on the FDG-FU scan, i.e., the survival cells and the new cells originating from the site regrowing. A VOI of about 100 ml was drawn in a region of the liver that was considered healthy on the basis of the FDG scans, CT scans, and MRI.

### Calculation of absorbed dose

The dosimetry was obtained using a previously validated method [30]: the ^90^Y-PET images were 3D, convolved with a dose deposition kernel directly providing the voxel-based dosimetry (in milligray per megabecquerel). Individual tumor-absorbed dose (in gray) was determined as the mean of the voxel-based dosimetry in the ^90^Y-PET VOI × the total injected activity.

### Determination of the early response

The tumor metabolic ratio at follow-up (MR) was calculated as:

(1) $$\text{MR}=\frac{({\text{SUV}}_{\mathrm{FU}}^{\mathrm{tum}}-{\text{SUV}}_{\mathrm{FU}}^{\mathrm{liv}})\times {\text{Vol}}_{\mathrm{FU}}^{\mathrm{tum}}}{({\text{SUV}}_{\mathrm{BL}}^{\mathrm{tum}}-{\text{SUV}}_{\mathrm{BL}}^{\mathrm{liv}})\times {\text{Vol}}_{\mathrm{BL}}^{\mathrm{tum}}}\times \frac{{\text{SUV}}_{\mathrm{BL}}^{\mathrm{liv}}}{{\text{SUV}}_{\mathrm{FU}}^{\mathrm{liv}}},$$

where SUV is the mean FDG standardized uptake value, and Vol is the volume of the VOI. Assuming that the metabolism of the tumor cells does not change too much between the FDG-BL and FDG-FU scans, the mean tumor SUV multiplied by the tumor volume is related to the number of living cells in the tumor. Two improvements were introduced in Equation 1: (1) subtraction of the mean liver SUV that takes into account the fact that, due to the replacement by normal liver cells and to the finite spatial resolution of the PET system, the measured mean SUV of a responding tumor may not be different than that of the liver; and (2) multiplication by the mean liver SUV inverse ratio that corrects, to some extent, for the variation of the patient glycemia between the FDG-BL and FDG-FU scans and for a possible metabolic boosting resulting from a general inflammation of the liver and hepatic metastasis as an effect of irradiation. Different variations of this tumor-to-liver ratio method were already used by others [35-37].

### Early dose-response analysis

The early dose-response relationship was fitted by minimizing:

(2) $${Χ}^{2}=\Sigma p,m{\left|SF_{p,m}-SF_{p,m}^{\mathrm{mod}}\right|}^{2},$$

where *m* is the index of the metastatic site in patient *p*, and SF is the survival fraction, i.e., the ratio between the number of living cells just after and before the therapy. SF is related to the metabolic ratio measured at follow-up by:

(3) $$SF_{p,m}=e^{-\ln(2)\delta t_{p}/DT}MR_{p,m},$$

where *δt*_*p*_ is the delay between the FDG-FU and FDG-BL scans, and DT is the tumor doubling time. The modeled survival fraction is:

(4) $$SF_{p,m}^{\mathrm{mod}}=e^{-\alpha HEF_{p}D_{p,m.}},$$

where *α* expresses the ability of one particle to induce a DNA double-strand break. HEF was assumed to be the same for all metastatic sites *m* in patient *p* and related to the Hb level (in gram per deciliter) according to:

(5) $$HEF_{p}=1+k(Hb_{p}-13)\text{.}$$

This results in a global fit of three parameters, DT (days), *α* (per gray), and *k* (deciliter per gram), on 25 points (number of metastatic sites), where DT and *α* were constrained to be positive.

HEF was introduced in Equation 4 in the similar way that the oxygen enhancement ratio (OER) was introduced for acute hypoxia [17,18]. Equation 5 was designed to give HEF = 1 for patients having a Hb level of 13 g/dL, which is a normal value. This is an arbitrary choice; a value other than 13 g/dL will just give other values for *α* and *k* but will give an identical fit quality.

## Results and discussion

### Results

**Figure 2:**
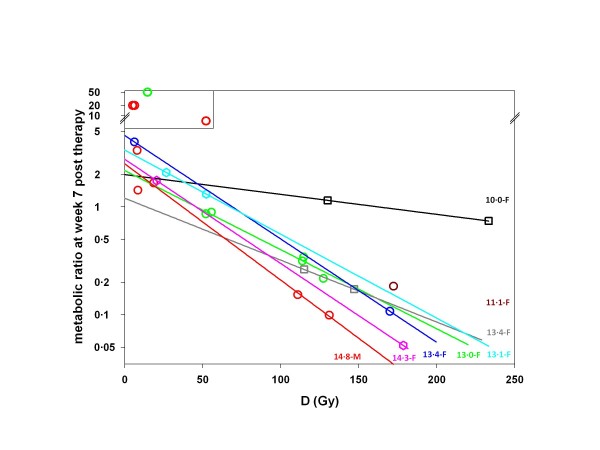
**Relationship between absorbed doses Dand tumor metabolic ratio (*MR*).** MR for the responding tumors are lower than 1. Each color refers to each patient (black - 1, blue - 2, brown - 3, red - 4, pink - 5, green - 6, cyan - 7, and grey - 8). Open circles and hexagons correspond to metastases from CRC, and open squares refer to metastases from melanoma. The patient gender and the Hb level (in gram per deciliter) on the day of the SIRT procedure are indicated at the extremity of each fitted line. Individual patient tumors were fitted by $gf\;e^{-\bar{\alpha }D}$, where *gf* is the growing fraction in the absence of an absorbed dose and $\bar{\alpha }$ is the overall cell radiosensitivity, i.e., containing the HEF factor. The box in the upper left corner identifies outliers (see ‘Results and discussion’). The lines are the patient data individually fitted with this single exponential.

Figure 2 shows the relationship between the absorbed doses and the response, estimated as the tumor metabolic ratio at approximately 7 weeks post-therapy for all metastatic sites. The tumor response was highly variable between patients but, per patient, was clearly dependent on the absorbed dose. Two patients showed a convincing response (gray squares and brown circle), one patient had two nonresponding tumors (cyan hexagons), whereas the remaining patients displayed a mix of responding and nonresponding tumors, depending on the absorbed doses.

All tumors show a trend towards a similar proliferation rate by extrapolation of the response for *D* = 0, except for four metastases that clearly behave as outliers (Figure 2, see box in the left upper corner). These four metastases belong to two patients with a long disease and therapy history (2 and 4 years). It is likely that these metastases may have acquired a phenotype resulting in a doubling time significantly shorter than the mean of the other metastases. The global fit is performed assuming that all the metastases have the same doubling time (DT, Equation 3); accordingly, these four metastases were considered to be outside the application domain of the model and were ruled out for the global fit.

**Figure 3:**
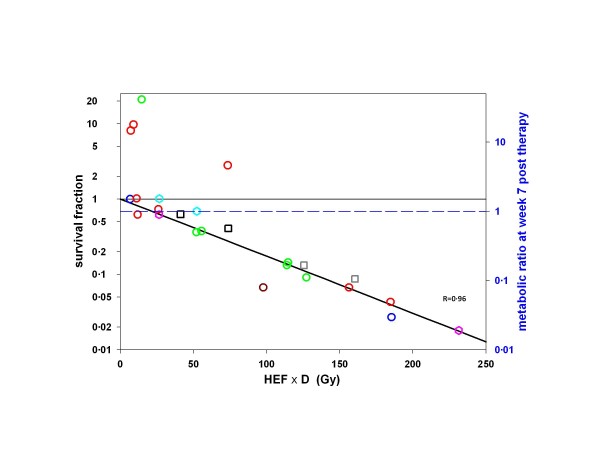
**Tumors survival fraction.** Relationship between absorbed dose D corrected by the HEF and the tumor cell survival fraction (left axis) and metabolic ratio (right axis) calculated at week 7 post-therapy using the doubling time obtained by the global fit. For colors and symbols refer to Figure 2. The line corresponds to Equation 4 with the parameters *α*, *k* and DT minimizing Equation 2, excluding the four metastases with SF > 1, which proved their shorter DT (using the actual metastasis DT in Equation 3 should necessarily give SF ≤ 1). These four metastases were already considered as outliers from Figure 2. See animation of the fit in Additional file 1.

The global fit, run to estimate the biological parameters, was therefore performed on the 21 remaining metastases using ΣPlot 2000 version 6.00 (Systat Software Inc, San Jose, CA, USA) and resulted in the following values (best parameter =  2.1*std error, i.e., 95% confidence interval): DT = 50   8 day, *α* = 0.018   0.003/Gy and *k* = 0.23   0.05 dL/g. The left axis in Figure 3 shows the tumor cell survival fraction (Equation 3) as a function of HEF_*p*_*D*_*p,m*_ that takes into account the Hb level (Equation 5). The line is the survival fraction modeled by Equation 4 using the parameters determined by the global fit (*R* = 0.96). It can be observed that a number of tumors showed a metabolic ratio higher than one (right axis) even though their survival fraction was lower than one. These tumors actually showed a response that is not sufficient to counterbalance at week 7 the regrowth of the viable tumor cells.

**Figure 4:**
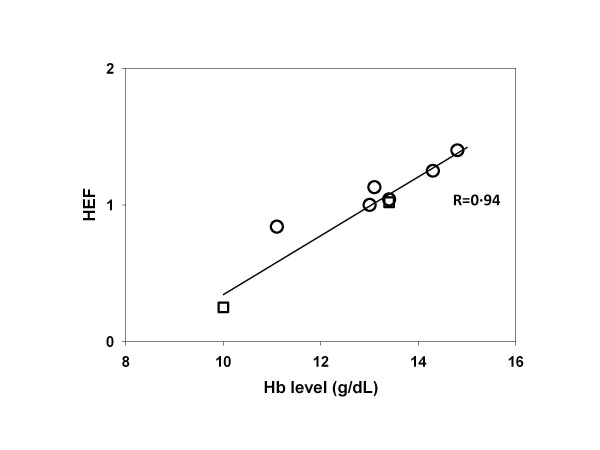
**Optimal values of HEF for each patient in relation with their Hb level.** Relationship between the Hb level and the optimal values of the HEF_*p*_ parameter obtained by individually fitting HEF for each patient dose-response using *α* and DT values determined from the global fit. The line corresponds to Equation 5, with *k* obtained by the global fit. *R* is the correlation coefficient obtained by making a linear regression of these optimal HEF_*p*_.

The optimal values of HEF for each patient in relation with their Hb level are shown in Figure 4. This graphical representation of Equation 5 indicates that the prediction of response in individual patients, taking into account their Hb level, is very close (*R* = 0.94) to what would be expected from the global fit (see Additional file 1).

### Discussion

This study clearly shows the impact of Hb level on tumor response. This impact was quantitatively interpreted, introducing a HEF, as the effect of tumor hypoxia induced by the patients' anemia (Figure 3). This is the first time that such analysis was applied to *in vivo* human data. This methodology is usually limited to preclinical models, such as cell assays or xenograft tumors in rodents. In preclinical models, almost all parameters are under control, such as the tumor cell line and time of progression. In the clinics, there is a large heterogeneity of metastases between patients but also within the same patient. In particular, the duration of progression of a single lesion in a particular patient is usually totally overlooked. This may lead to variations in the tumor cell phenotype, with some metastases becoming significantly more proliferative. We initially assessed tumor metabolic response using FDG uptake without knowledge of any other parameters. As already observed using morphological estimates, 50 Gy was required to observe any response. There was a clear trend towards a dose-response relationship, but the correlation was weak (*R* = 0.51). After observing the different response patterns between patients, we looked at the Hb level as a possible factor accounting for this variance. After individually fitting patient data and highlighting the Hb level in Figure 2, it appeared obvious that some relationship did exist. We therefore applied the concept of a radiosensitivity modulation to the data, a paradigm that is classically utilized for *in vitro* data, and that, to the best of our knowledge, has never been applied to internal radiotherapy of patients.

This correction factor appeared tightly related to the Hb level and, hence, was called HEF. For Hb level ranging between 10 and 15 g/dL, the individual HEF estimate displayed a linear increase, reaΧng a ratio of 5.6 between these two extreme Hb levels (Figure 4). This is in keeping with the major impact of Hb level observed for EBRT in recurrent rectal cancer [38]. This is also in line with the observation of Vaupel et al. [9] showing by an invasive method that the tumor pO_2_ can vary by a factor 4 in breast cancer as a function of the Hb level. Such linear relationship is however not the rule: in uterine and cervical cancer and in head and neck cancer, a bell-shaped relationship was reported by Vaupel et al. [9], with a maximal pO_2_ value measured for Hb levels of 12.5 and 14 g/dL, respectively. Such intraoperative pO_2_ measurements are lacking for hepatic metastases from CRC.

The radiation sensitivity parameter α found (0.018/Gy for a normal Hb level of 13 g/dL) is about 15 × lower than values found in cell assays [39]: 0.31/Gy for melanoma and 0.25/Gy for CRC. However, this is consistent with the comparison of *in vivo* xenograft and *in vitro* data that suggests that the survival is considerably higher *in vivo*[40]. In a retrospective analysis of SIRT in 73 patients with hepatocellular carcinoma, Strigari et al., using a radiobiological model of tumor control probability (TCP) assessed in CT scan by the RECIST criteria but without addressing anemia, found two very low extreme bounds for the value of α [41]: 0.001 and 0.005/Gy, much lower than values reported using cell assays [42]: 0.1 and 0.4/Gy for HepG2 and Hep3b cell lines, respectively. The factor 5 between the extreme bounds of the radiosensitivity found by Strigari et al. is similar to the factor 5.6 that we found between patients with extreme Hb levels.

Kato et al. [43] set up an elegant experiment that enlightens the observations listed in both previous paragraphs. They modeled chronic hypoxia, which is usually the rule in patients, by culturing gastric cancer cells (OCUM-12 line) in successively decreasing partial oxygen pressures (pO2). An important fraction of cells died, but the survivors resulted in an OCUM-12 line that was still able to proliferate at 13 mmHg pO2 (called OCUM-12/hypo). The fit of the survival curves they measured shows that the chronic 13 mmHg pO2 exposure reduced by a factor 14 the radiosensitivity *α* of the OCUM-12/hypo line as compared to that of the original OCUM-12 parent line set in acute 13 mmHg pO2 for 24 h. For comparison, acute 13 mmHg pO2 in most cell lines decreases the radiosensitivity by only a factor of about 1.2 versus normoxic conditions ([44], Figure 3). Lastly, the results of Vaupel et al. [9] show that, for a normal Hb level of 13 g/dL, the typical tumor pO2 is in fact about 13 mmHg due to the disturbed vascular arΧtecture.

In a previous study on ^90^Y-DOTATOC therapy in patients with neuroendocrine tumors [45], we have shown for the first time a good relationship between tumor shrinkage and absorbed dose using morphological imaging. There was, however, considerable spread in this dose-response relationship. In the current study, the correlation is excellent (*R* = 0.96) and with little spread. This can be explained by the presence of more favorable features in the current study: (1) the absorbed dose is directly assessed from the distribution of the therapeutic agent, (2) the effective half-life is assumed identical to that of the physical decay and thus known with high accuracy, and (3) the Hb enhancement effect was taken into consideration.

In this study, the mean absorbed dose modulated by the Hb level proved being sufficient to correlate to the tumor metabolic response. Using this mean absorbed dose, the cell killing is overestimated in the regions of the VOI that received lower dose, but is underestimated elsewhere. Both effects tend to average. Further refinement to account for the dose distribution in the VOI, such as using the equivalent uniform dose (EUD), will also require the assessment of living cell distribution in this VOI. This could be done using the base‐line FDG scan. However, due to tumor evolution, the delay in our study between the base‐line FDG and the ^90^Y-microsphere scans makes a tumor co-registration on a voxel basis between the two scans impossible.

Conceptually, the doubling time could be assessed by extrapolating for each patient the absorbed dose-metabolic ratio relationship to the left axis (i.e., *D* = 0), as shown in Figure 2. However, this method is not applicable in view of the following: (1) the doubling time is not only patient dependent but also metastasis dependent (illustrated by the red and green circles), and (2) some patients have only one tumor (brown circle) or only two metastases far away from the left axis (gray squares), making the extrapolation impossible or very unstable, respectively. Accordingly, a single doubling time value common to all the metastasis was used in the global fit, and the value found (50 days) is in good agreement with CEA doubling time measured in 33 patients with hepatic CRC metastases (47   25 days) [46]. Individually fitting the four outlier metastases using Equations 3 and 4 with *α* = 0 allows giving an upper bound for their doubling time that was found to be 22.5, 13.9, 13.7 and 11.2 days, respectively. In a prospective clinical study, the individual metastasis doubling time could be assessed by acquiring an additional FDG scan close to the ^90^Y-PET scan, the FDG-BL scan being usually performed a few weeks before the SIRT procedure. This procedure should also help to investigate whether a lag time before regrowth starts after irradiation should be introduced in Equation 3, such as proposed in other models [47].

Our results showed that a higher Hb level is important to get an optimal tumor response in ^90^Y SIRT. Various correction methods to overcome tumor hypoxia in EBRT are under clinical investigation and intensively reviewed [21-26]: red cell transfusion or the use of recombinant human erythropoietin to correct anemia, enriched oxygen breathing or hyperbaric condition to increase tumor oxygenation, and the use of radiosensitizing drugs. Although some strategies are currently being proposed, contradictory results still exist between clinical studies. These studies are based on the late assessment of the disease outcome: local tumor control using morphological measurements (e.g., CT scan) or patient cumulative survival curves. Of course, these clinical studies are highly demanded because they really assess the final purpose of the therapy. However, they require large patient series and long follow-up period. Up to now, the quantitative analysis of the relationship between SF and absorbed dose has been widely performed on *in vitro* assays. Similar studies in rodent models allow obtaining this information on an individual tumor cell line in a small animal series. The same paradigm applied here allows, in individual patient, assessment of the effect of hypoxia on several tumors. This may, in turn, be used to evaluate potential optimization protocols in small patient series.

## Conclusions

The current results show that absorbed dose alone is not sufficient to predict early tumor response, but this become feasible when introducing a simple hemoglobin enhancement factor linear to the patient's Hb level. While the oxygen effect is an important field of research in EBRT, these results support the study of similar strategies to reduce hypoxia in the framework of internal radiotherapy. The quantitative analysis of the relationship between tumor-absorbed doses and early response has the power to allow fast screening of hypoxia correction methods in limited patient series. Internal radiotherapy may be more efficient if performed earlier in the therapy line, when anemia related to disease progression or to treatment remains limited.

## Competing interests

The authors declare that they have no competing interests.

## Authors’ contribution

SW elaborated the Hb-dependent dosimetry model and performed the results analysis. RL and FJ performed the study design. RL, PG, and MVE carried out the treatment and follow-up of the patients. SW, RL, SP, and FJ participated in the paper writing. All authors read and approved the final manuscript.

## Supplementary Material

Additional file 1 Hemoglobin level significantly impacts the tumor cell survival fraction in humans after internal radiotherapy: application of a preclinical radiobiology model to clinical data. Click here for file

## References

[B1] Caro JJ Salas M Ward A Goss G Anemia as an independent prognostic factor for survival in patients with cancer. A systemic quantitative review Cancer 2001 91 2214 2221 10.1002/1097-0142(20010615)91:12<2214::AID-CNCR1251>3.0.CO;2-P 11413508

[B2] Harrison LB Shasha D Homel P Prevalence of anemia in cancer patients undergoing radiotherapy: prognostic significance and treatment Oncology 2002 63 11 18 10.1159/000067147 12466640

[B3] Harrison LB Chadha M Hill RJ Hu K Shasha D Impact of tumour hypoxia and anemia on radiation therapy outcomes Oncologist 2002 7 492 508 10.1634/theoncologist.7-6-492 12490737

[B4] Nowrousian MR Nowrousian MR Prevalence, pathophysiology, predictive factors, and prognostic significance of anemia in cancer chemotherapy Recombinant human erythropoietin (rhEPO) in clinical oncology 2002 2 Springer, Wien, New York 63 100

[B5] Van Belle SJ-P Cocquyt V Impact of haemoglobin levels on the outcome of cancers treated with chemotherapy Crit Rev Oncol Hematol 2003 47 1 11 10.1016/S1040-8428(03)00093-3 12853095

[B6] Clarke H Pallister CJ The impact of anaemia on outcome in cancer Clin Lab Haem 2005 27 1 13 10.1111/j.1365-2257.2004.00664.x 15686502

[B7] Österborg A Bokemeyer C, Ludwig H Anaemia in patients with cancer: association to prognosis and prediction of response to erythropoietic agents Anaemia in Cancer 2005 Elsevier, Edinburgh London New York

[B8] Vaupel P Mayer A Hypoxia in cancer: significance and impact on clinical outcome Cancer Metastasis Rev 2007 26 2 225 239 10.1007/s10555-007-9055-1 17440684

[B9] Vaupel P Mayer A Höckel M Nowrousian MR Relationship between hemoglobin levels and tumour oxygenation Recombinant human erythropoietin (rhEPO) in clinical oncology 2008 Springer, Wien, New York

[B10] Höckel M Vaupel P Tumour hypoxia: definitions and current clinical, biologic, and molecular aspects J Natl Cancer Inst 2001 93 266 276 10.1093/jnci/93.4.266 11181773

[B11] Semenza GL Hypoxia, clonal selection, and the role of HIF-1 in tumour progression Crit Rev Biochem Mol Biol 2000 35 71 103 10.1080/10409230091169186 10821478

[B12] Semenza GL Involvement of hypoxia-inducible factor 1 in human cancer Intern Med 2002 41 79 83 10.2169/internalmedicine.41.79 11868612

[B13] Semenza GL HIF-1 and tumour progression: pathophysiology and therapeutics Trends Mol Med 2002 8 suppl 1 S62 S67 11927290 10.1016/s1471-4914(02)02317-1

[B14] Harris AL Hypoxia—a key regulatory factor in tumour growth Nat Rev Cancer 2002 2 38 47 10.1038/nrc704 11902584

[B15] Leo C Giaccia AJ Denko NC The hypoxic tumour microenvironment and gene expression Semin Radiat Oncol 2004 14 207 214 10.1016/j.semradonc.2004.04.007 15254863

[B16] Stabin MG Fundamentals of nuclear medicine dosimetry 2008 Springer, New York

[B17] Beyzadeoglu M Ozyigit G Ebruli C Radiobiology Basic radiation oncology 2010 Springer, New York

[B18] McParland BJ Biological effects of ionizing radiation Nuclear Medicine Radiation Dosimetry 2010 Springer, New York

[B19] van der Poel HG Antonini N Hoefnagel CA Horenblas S Valdes Olmos RA Serum hemoglobin levels predict response to strontium-89 and rhenium-186-HEDP radionuclide treatment for painful osseous metastases in prostate cancer Urol Int 2006 77 1 50 56 10.1159/000092935 16825816

[B20] Windsor PM Predictors of response to strontium-89 (Metastron) in skeletal metastases from prostate cancer: report of a single centre's 10-year experience: palliation and metastatic disease Clin Oncol 2001 13 3 219 227 10.1053/clon.2001.9257 11527299

[B21] Wardman P Chemical radiosensitizers for use in radiotherapy Clin Oncol 2007 19 6 397 417 10.1016/j.clon.2007.03.010 17478086

[B22] Ahn GO Brown JM Combinations of hypoxia-targeting compounds and radiation-activated prodrugs with ionizing radiation Multimodal concepts for integration of cytotoxic drugs and radiation therapy 2006 Springer, New York 67 91 [Brown MJ, Nieder C, Brady LW (Series Editors): Medical Radiology]

[B23] Janssen HL Haustermans KM Balm AJ Begg AC Hypoxia in head and neck cancer: how much, how important? Head Neck 2005 27 7 622 638 10.1002/hed.20223 15952198

[B24] Hu K Harrison LB Impact of anemia in patients with head and neck cancer treated with radiation therapy Curr Treat Options Oncol 2005 6 1 31 45 10.1007/s11864-005-0011-4 15610713

[B25] Ozsahin M Azria D Beer K Mirimanoff RO Zouhair A External radiotherapy and anaemia treatment: state of the art Swiss Med Wkly 2005 135 1–2 4 10 15662573 10.4414/smw.2005.10805

[B26] Horsman MR van der Kogel AJ Joiner M, Kogel A Therapeutic approaches to tumour hypoxia Basic clinical radiobiology 2009 MPG Books, London

[B27] Lhommel R Goffette P Van den Eynde M Jamar F Pauwels S Bilbao JI Walrand S Yttrium-90 TOF PET scan demonstrates high-resolution biodistribution after liver SIRT Eur J Nucl Med Mol Imaging 2009 36 10 1969 10.1007/s00259-009-1210-1 19618182

[B28] Walrand S Hesse S Demonceau G Pauwels S Jamar F Yttrium-90 labeled microspheres tracking during liver selective internal radiotherapy by bremsstrahlung pinhole SPECT: feasibility study and evaluation in an abdominal phantom EJNMMI Res 2011 1 32 10.1186/2191-219X-1-32 22214246 PMC3377914

[B29] Werner MK Brechtel K Beyer T Dittmann H Pfannenberg C Kupferschläger J PET/CT for the assessment and quantification of 90Y biodistribution after selective internal radiotherapy (SIRT) of liver metastases Eur J Nucl Med Mol Imaging 2010 37 2 407 408 10.1007/s00259-009-1317-4 19997914

[B30] Lhommel R van Elmbt L Goffette P Van den Eynde M Jamar F Pauwels S Walrand S Feasibility of yttrium-90 TOF-PET based dosimetry in liver metastasis therapy using SIR-spheres Eur J Nucl Med Mol Imaging 2010 37 9 1654 1662 10.1007/s00259-010-1470-9 20422185

[B31] van Elmbt L Vandenberghe S Walrand S Pauwels S Jamar F Comparison of yttrium-90 quantitative imaging by TOF and non-TOF PET in a phantom of liver selective internal radiotherapy Phys Med Biol 2011 56 21 6759 6777 10.1088/0031-9155/56/21/001 21970976

[B32] Walrand S Flux GD Konijnenberg MW Valkema R Krenning EP Lhommel R Pauwels S Jamar F Dosimetry of yttrium-labelled radiopharmaceuticals for internal therapy: (86)Y or (90)Y imaging? Eur J Nucl Med Mol Imaging 2011 38 1 suppl 1 S57 S68 21484382 10.1007/s00259-011-1771-7

[B33] Garin E Lenoir L Rolland Y Edeline J Mesbah H Laffont S Porè P Clegravement B Raoul JL Boucher E Dosimetry based on 99mTc-¯oaggregated albumin SPECT/CT accurately predicts tumor response and survival in hepatocellular carcinoma patients treated with 90Y-loaded glass microspheres: preliminary results J Nucl Med 2012 53 2 255 263 10.2967/jnumed.111.094235 22302962

[B34] Kennedy A Nag S Salem R Murthy R McEwan AJ Nutting C Benson A Benson A Espat J Bilbao JI Sharma RA Thomas JP Coldwell D Recommendations for radioembolization of hepatic malignancies using yttrium-90 microsphere brachytherapy: a consensus panel report from the radioembolization brachytherapy oncology consortium Int J Radiat Oncol Biol Phys 2007 68 1 13 23 10.1016/j.ijrobp.2006.11.060 17448867

[B35] Findlay M Young H Cunningham D Iveson A Cronin B Hickish T Pratt B Husband J Flower M Ott R Noninvasive monitoring of tumour metabolism using fluorodeoxyglucose and positron emission tomography in colorectal cancer liver metastases: correlation with tumour response to fluorouracil J Clin Oncol 1996 14 700 708 8622014 10.1200/JCO.1996.14.3.700

[B36] Flamen P Van Cutsem E Lerut A Cambier JP Haustermans K Bormans G De Leyn P Van Raemdonck D De Wever W Ectors N Maes A Mortelmans L Positron emission tomography for assessment of the response to induction radiochemotherapy in locally advanced oesophageal cancer Ann Oncol 2002 13 3 361 368 10.1093/annonc/mdf081 11996465

[B37] Mackie GC Shulkin BL Ribeiro RC Worden FP Gauger PG Mody RJ Connolly LP Kunter G Rodriguez-Galindo C Wallis JW Hurwitz CA Schteingart DE Use of [18F]fluorodeoxyglucose positron emission tomography in evaluating locally recurrent and metastatic adrenocortical carcinoma J Clin Endocrinol Metab 2006 91 7 2665 2671 10.1210/jc.2005-2612 16621901

[B38] Rades D Kuhn H Schultze J Homann N Brandenburg B Schulte R Krull A SΧld SE Dunst J Prognostic factors affecting locally recurrent rectal cancer and clinical significance of hemoglobin Int J Radiat Oncol Biol Phys 2008 70 4 1087 1093 10.1016/j.ijrobp.2007.07.2364 17892921

[B39] Fertil B Malaise EP Intrinsic radiosensitivity of human cell lines is correlated with radioresponsiveness of human tumours: analysis of 101 published survival curves Int J Radiat Oncol Biol Phys 1985 11 9 1699 1707 10.1016/0360-3016(85)90223-8 4030437

[B40] Malaise EP Fertil B Chavaudra N Guichard M Distribution of radiation sensitivities for human tumour cells of specific histological types: comparison of in vitro to in vivo data Rad Oncology Biol Phys 1986 12 617 624 10.1016/0360-3016(86)90071-4 3009370

[B41] Strigari L Sciuto R Rea S Carpanese L Pizzi G Soriani A Iaccarino G Benassi M Ettorre GM Maini CL Efficacy and toxicity related to treatment of hepatocellular carcinoma with 90Y-SIR spheres: radiobiologic considerations J Nucl Med 2010 51 9 1377 1385 10.2967/jnumed.110.075861 20720056

[B42] Zheng XK Chen LH Yan X Wang HM Impact of prolonged fraction dose-delivery time modeling intensity-modulated radiation therapy on hepatocellular carcinoma cell killing World J Gastroenterol 2005 11 10 1452 1456 15770720 10.3748/wjg.v11.i10.1452 PMC4305686

[B43] Kato Y Yashiro M Fuyuhiro Y Kashiwagi S Matsuoka J Hirakawa T Noda S Aomatsu N Hasegawa T Matsuzaki T Sawada T Ohira M Hirakawa K Effects of acute and chronic hypoxia on the radiosensitivity of gastric and esophageal cancer cells Anticancer Res 2011 31 3369 3376 21965748

[B44] Carlson DJ Keall PJ Loo BW Chen ZJ Brown JM Hypofractionation results in reduced tumour cell kill compared to conventional fractionation for tumours with regions of hypoxia Int J Radiation Oncology Biol Phys 2011 79 4 1188 1195 10.1016/j.ijrobp.2010.10.007 PMC3053128 21183291

[B45] Barone R Borson-Chazot F Valkema R Walrand S Chauvin F Gogou L Kvols LK Krenning EP Jamar F Pauwels S Patient-specific dosimetry in predicting renal toxicity with (90)Y-DOTATOC: relevance of kidney volume and dose rate in finding a dose-effect relationship J Nucl Med 2005 46 suppl 1 99S 106S 15653658

[B46] Staab HJ Anderer FA Hornung A Stumpf E Fischer R Doubling time of circulating CEA and its relation to survival of patients with recurrent colorectal cancer Br J Cancer 1982 46 5 773 781 10.1038/bjc.1982.270 7171456 PMC2011150

[B47] Fowler JF The linear-quadratic formula and progress in fractionated radiotherapy Br J Radiol 1989 62 679 694 10.1259/0007-1285-62-740-679 2670032

