# Supplementary material for: Hemoglobin level significantly impacts the tumor cell survival fraction in humans after internal radiotherapy
Source: EJNMMI Res. 2012 May 19;2:20. doi: 10.1186/2191-219X-2-20 (PMC3413597; doi:10.1186/2191-219X-2-20)
Supplement: Additional file 1 — Hemoglobin level significantly impacts the tumor cell survival fraction in humans after internal radiotherapy: application of a preclinical radiobiology model to clinical data. [file 2191-219X-2-20-S1.ppt]

## Slide 1
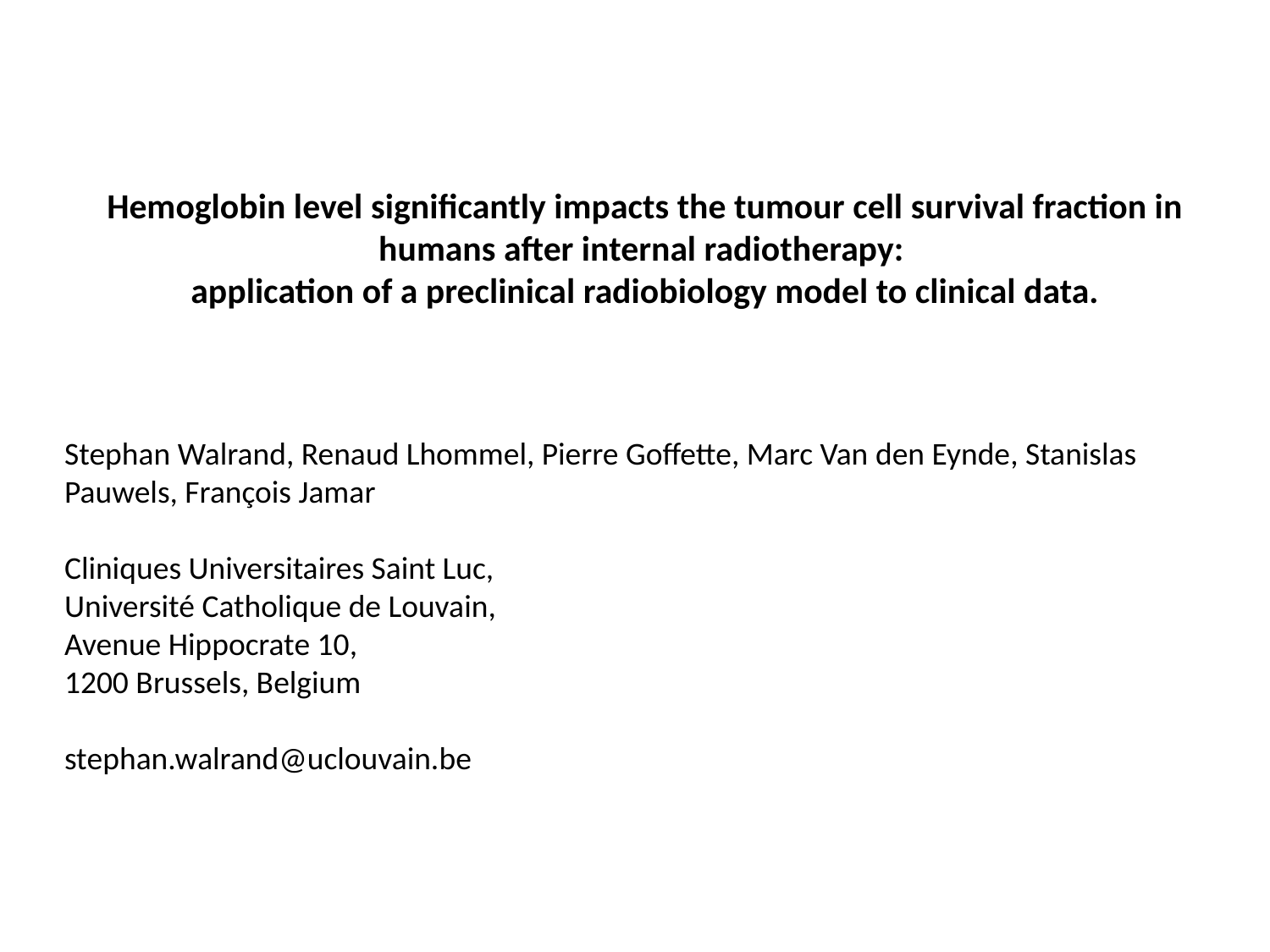

Hemoglobin level significantly impacts the tumour cell survival fraction in humans after internal radiotherapy:
application of a preclinical radiobiology model to clinical data.
Stephan Walrand, Renaud Lhommel, Pierre Goffette, Marc Van den Eynde, Stanislas Pauwels, François Jamar
Cliniques Universitaires Saint Luc,
Université Catholique de Louvain,
Avenue Hippocrate 10,
1200 Brussels, Belgium
stephan.walrand@uclouvain.be

## Slide 2
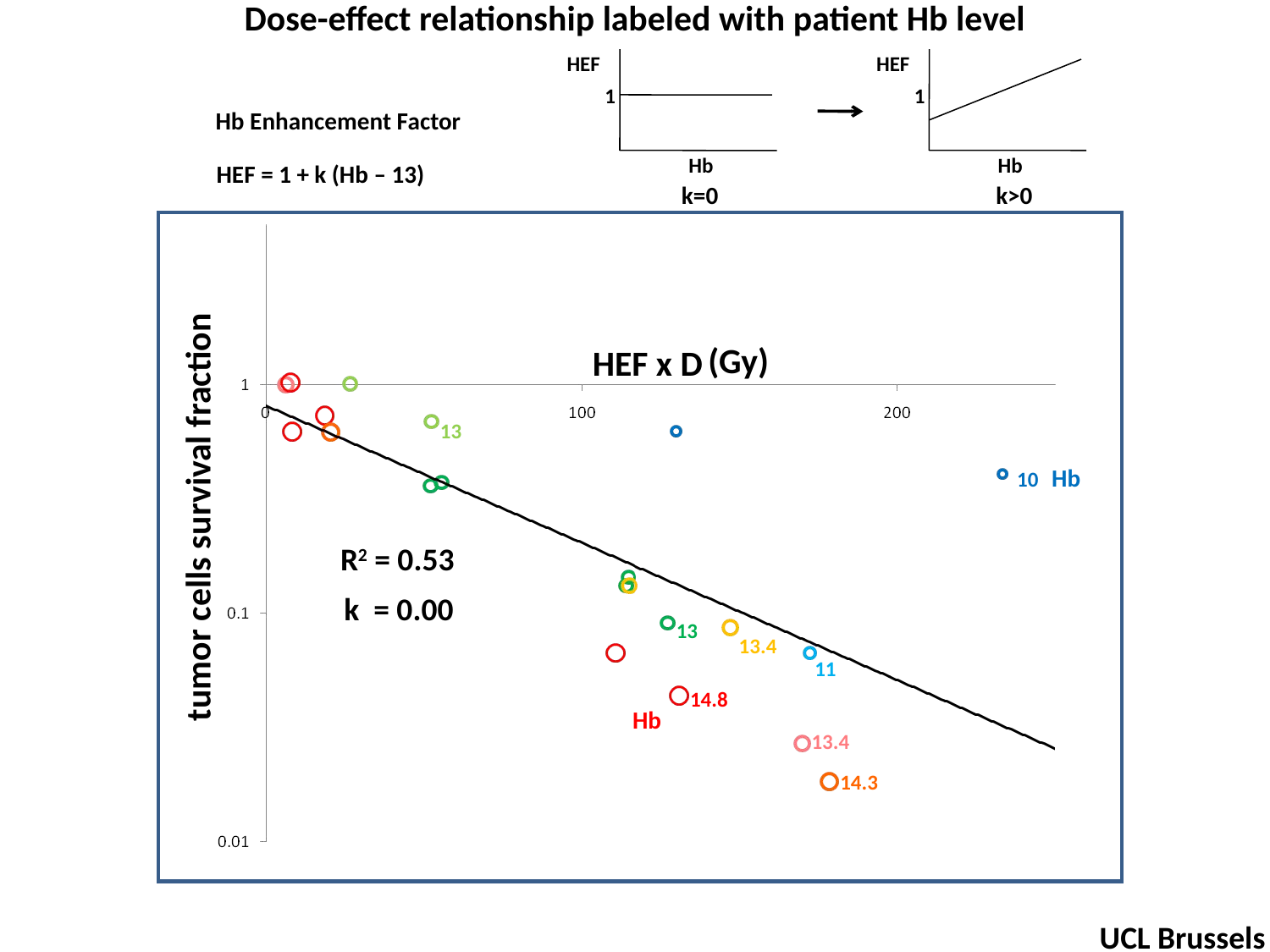

Dose-effect relationship labeled with patient Hb level
HEF
1
Hb
k=0
k = 0.00
HEF
1
Hb
k>0
Hb Enhancement Factor
HEF = 1 + k (Hb – 13)
OER
13
10
R2 = 0.53
13
13.4
11
14.8
13.4
14.3
(Gy)
HEF x D
Hb
tumor cells survival fraction
Hb
let see in the following animation
what will happen if we increase k
UCL Brussels

## Slide 3
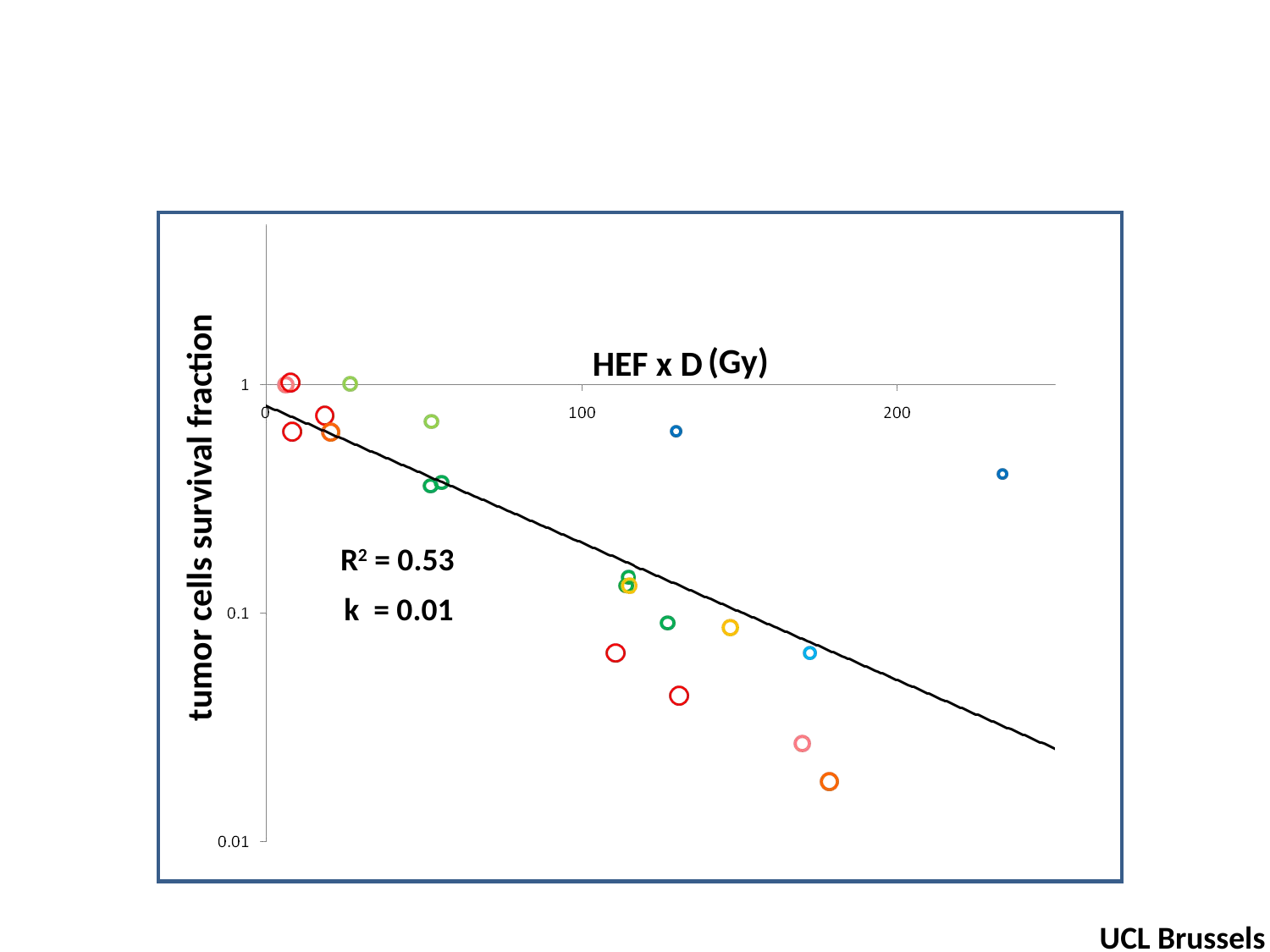

(Gy)
tumor cells survival fraction
R2 = 0.53
-
HEF x D
k = 0.01
UCL Brussels

## Slide 4
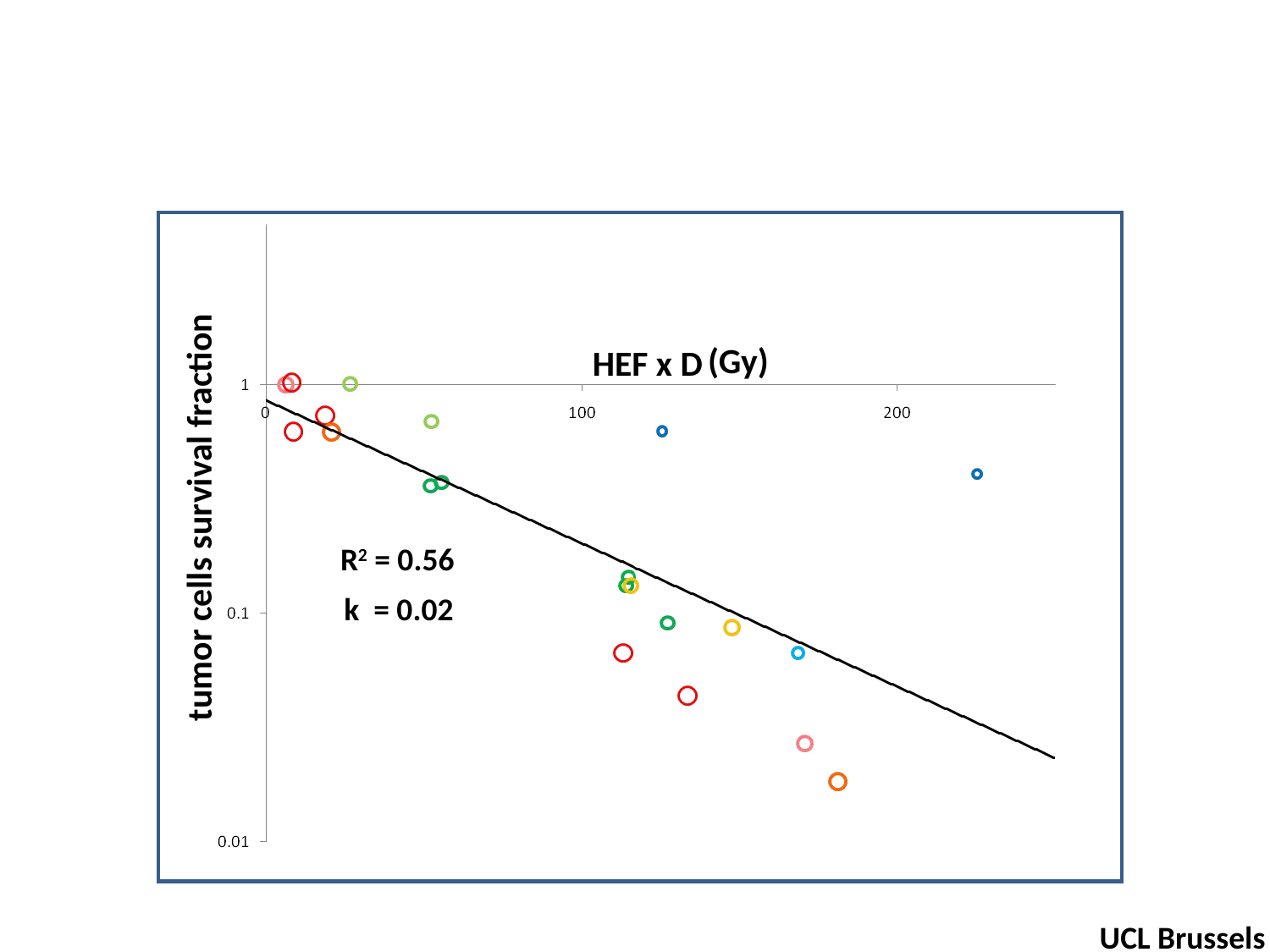

(Gy)
tumor cells survival fraction
R2 = 0.56
HEF x D
k = 0.02
UCL Brussels

## Slide 5
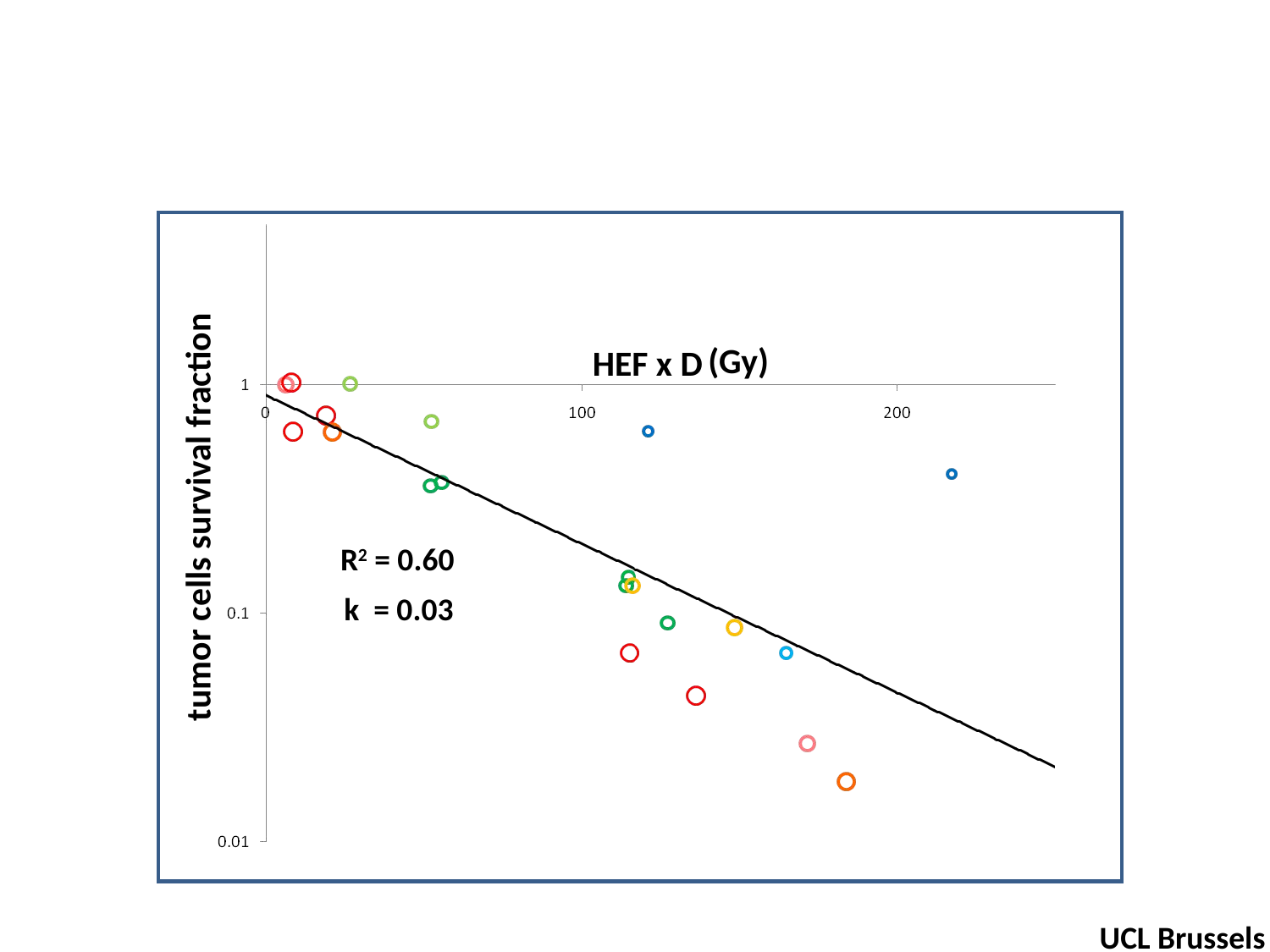

R2 = 0.60
(Gy)
tumor cells survival fraction
HEF x D
k = 0.03
UCL Brussels

## Slide 6
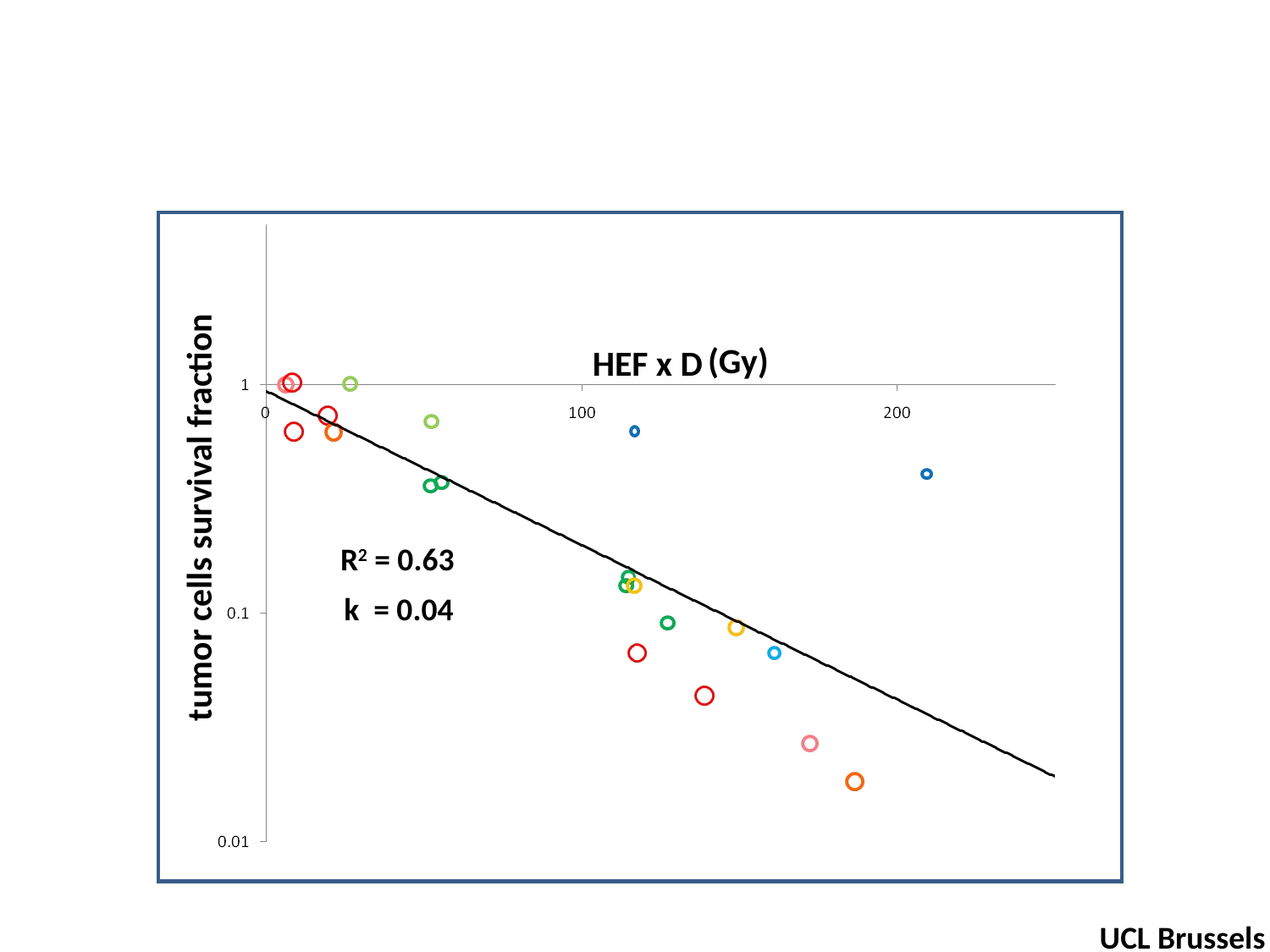

(Gy)
tumor cells survival fraction
R2 = 0.63
HEF x D
k = 0.04
UCL Brussels

## Slide 7
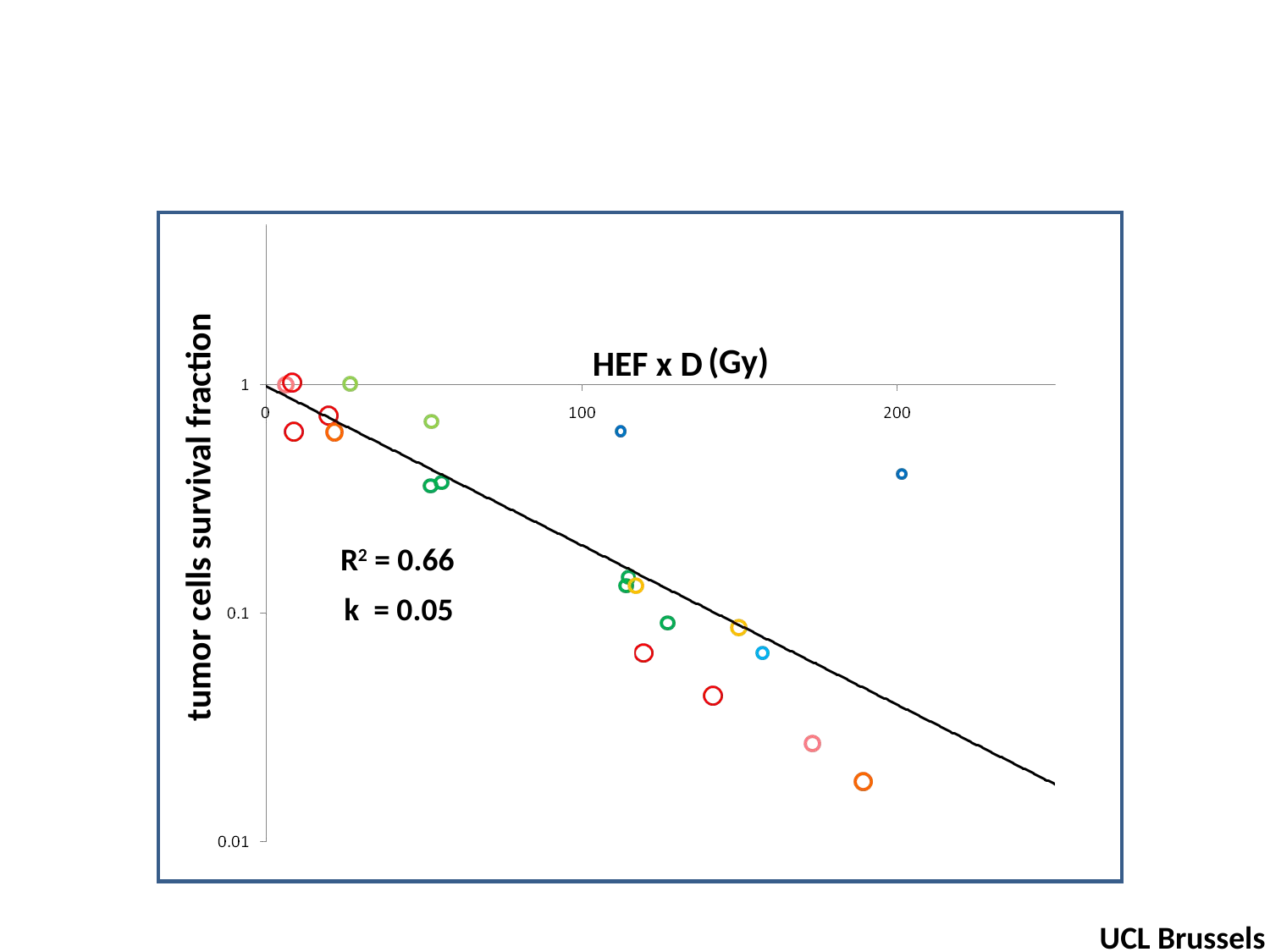

R2 = 0.66
(Gy)
tumor cells survival fraction
HEF x D
k = 0.05
UCL Brussels

## Slide 8
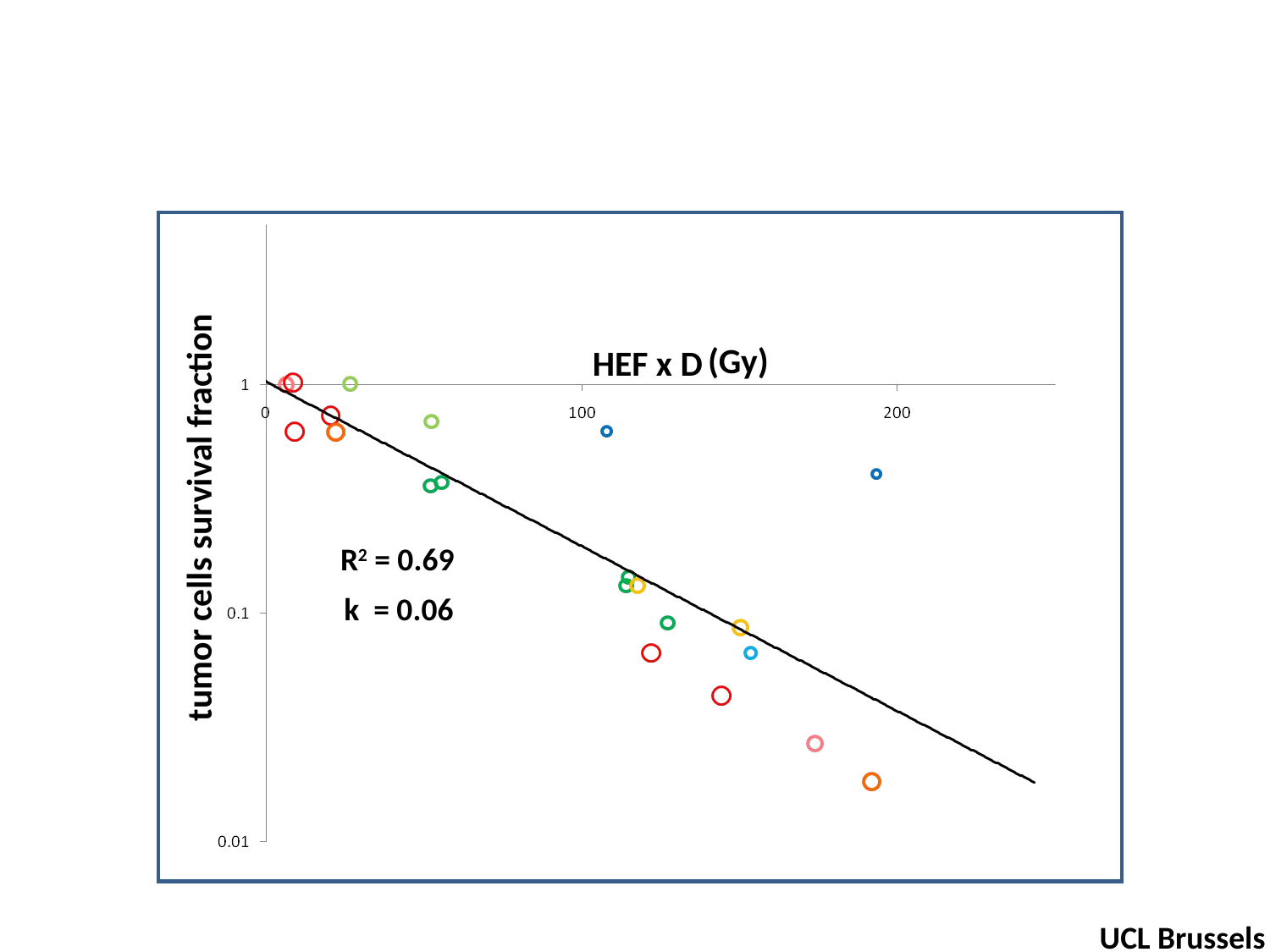

(Gy)
tumor cells survival fraction
R2 = 0.69
HEF x D
k = 0.06
UCL Brussels

## Slide 9
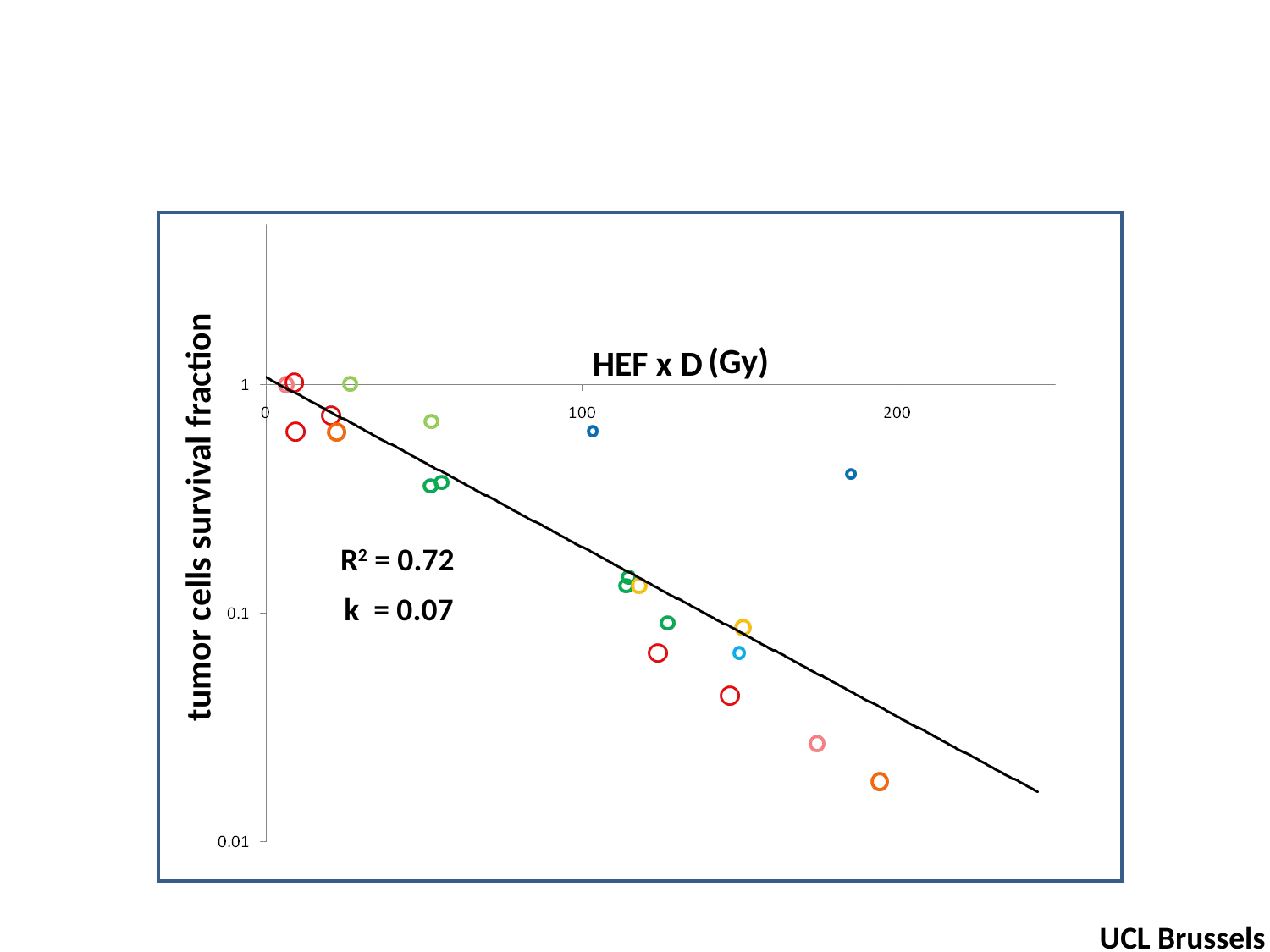

R2 = 0.72
(Gy)
tumor cells survival fraction
HEF x D
k = 0.07
UCL Brussels

## Slide 10
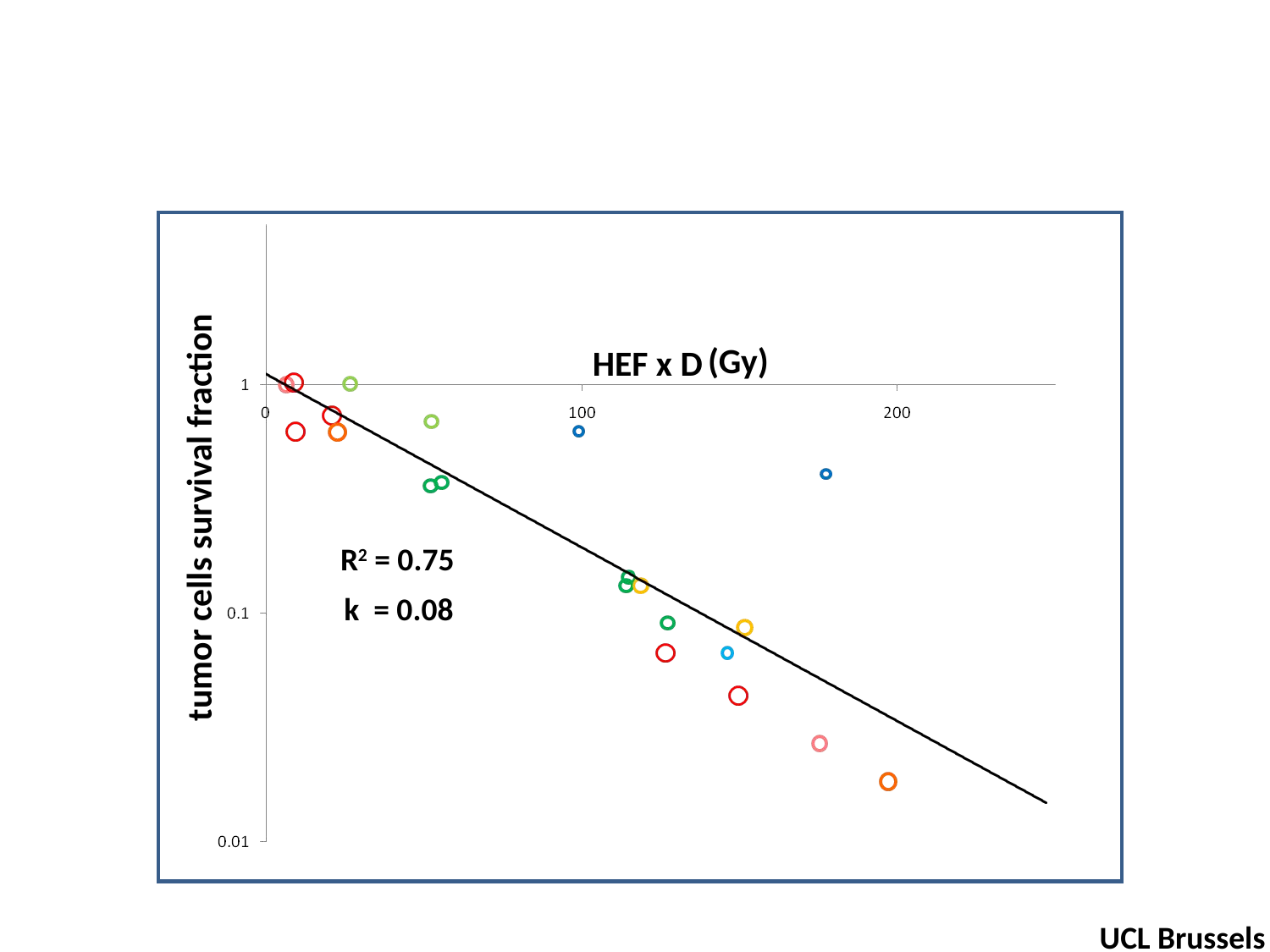

(Gy)
tumor cells survival fraction
R2 = 0.75
HEF x D
k = 0.08
UCL Brussels

## Slide 11
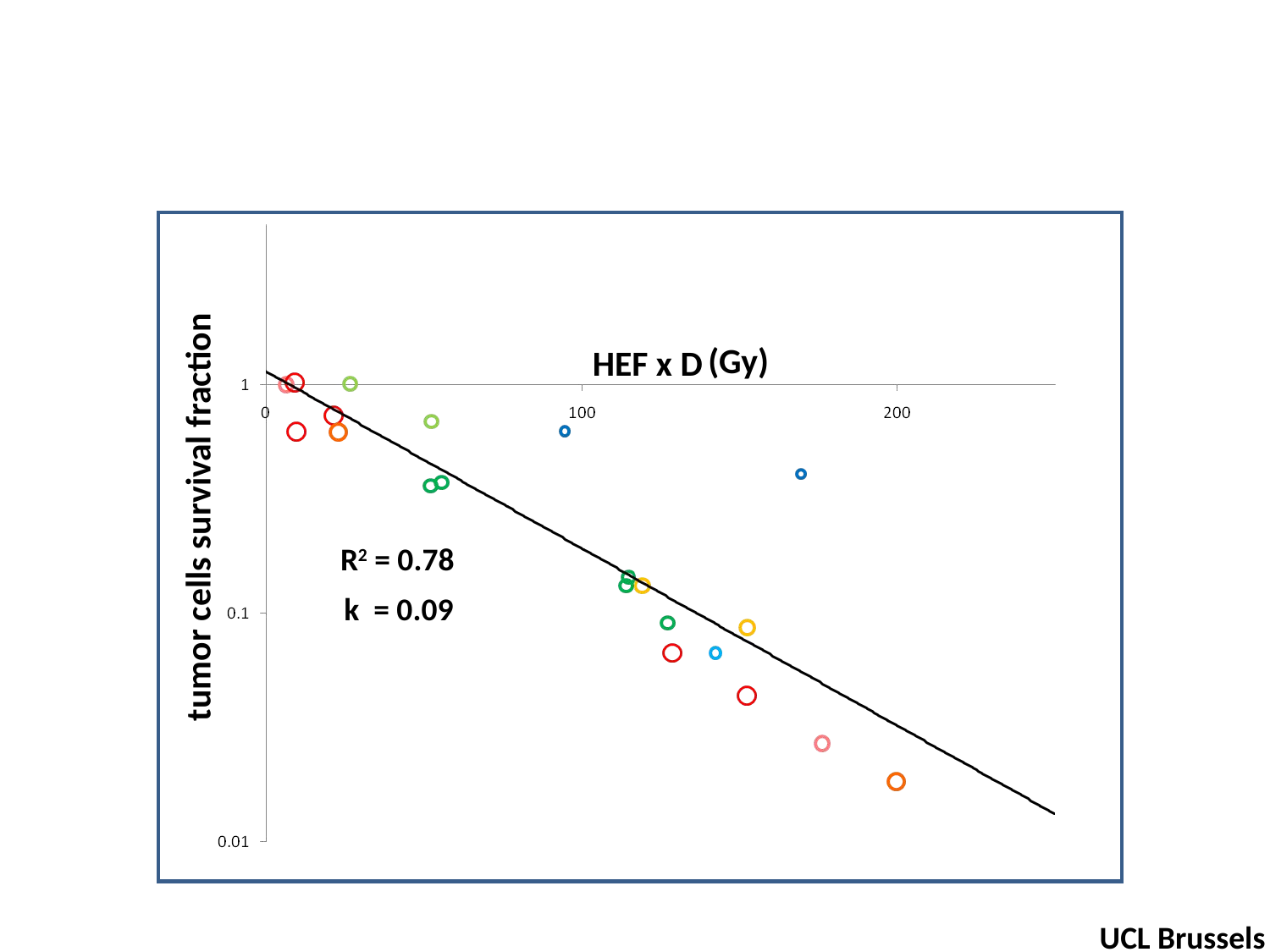

R2 = 0.78
(Gy)
tumor cells survival fraction
HEF x D
k = 0.09
UCL Brussels

## Slide 12
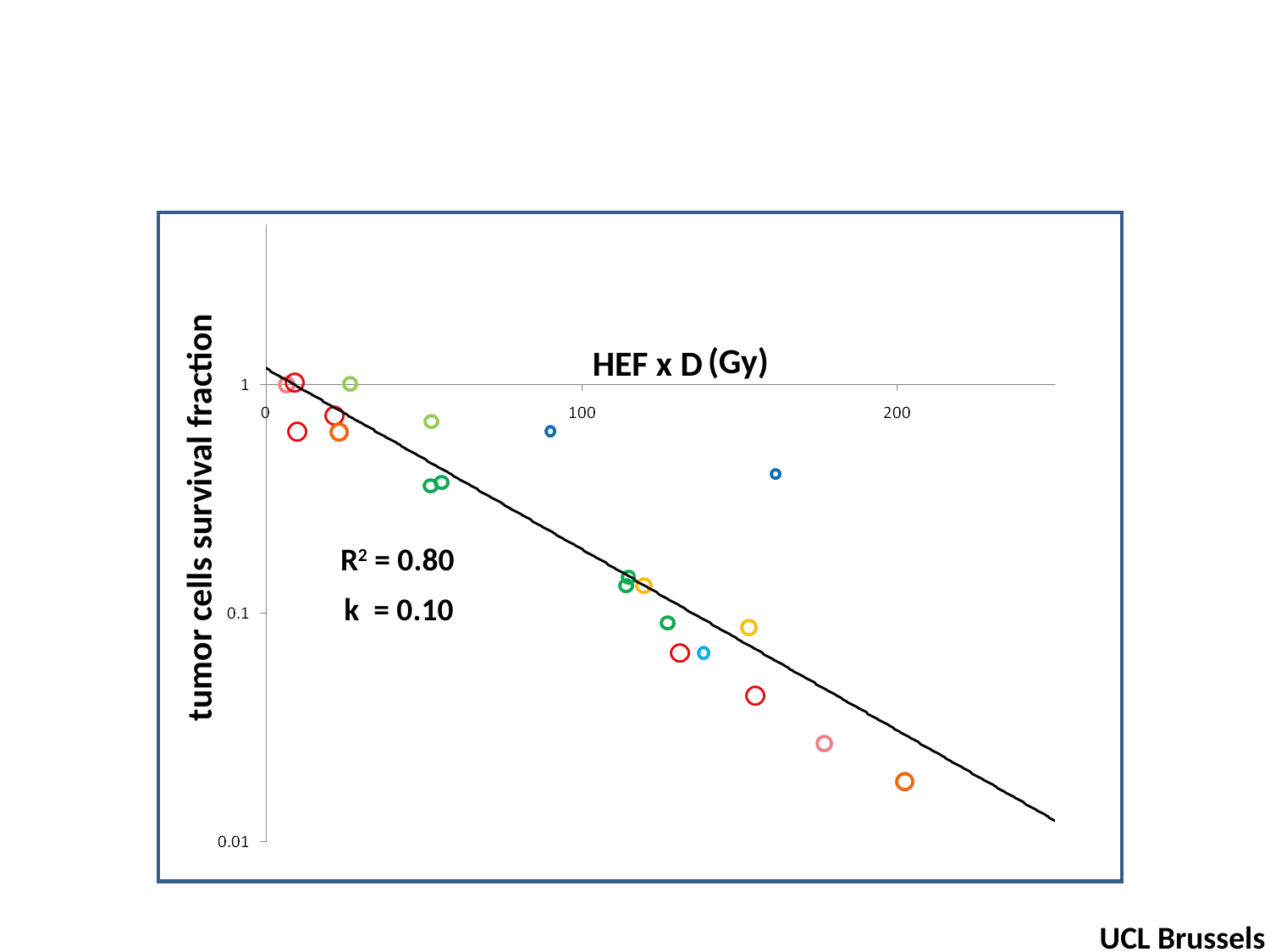

(Gy)
tumor cells survival fraction
R2 = 0.80
HEF x D
k = 0.10
UCL Brussels

## Slide 13
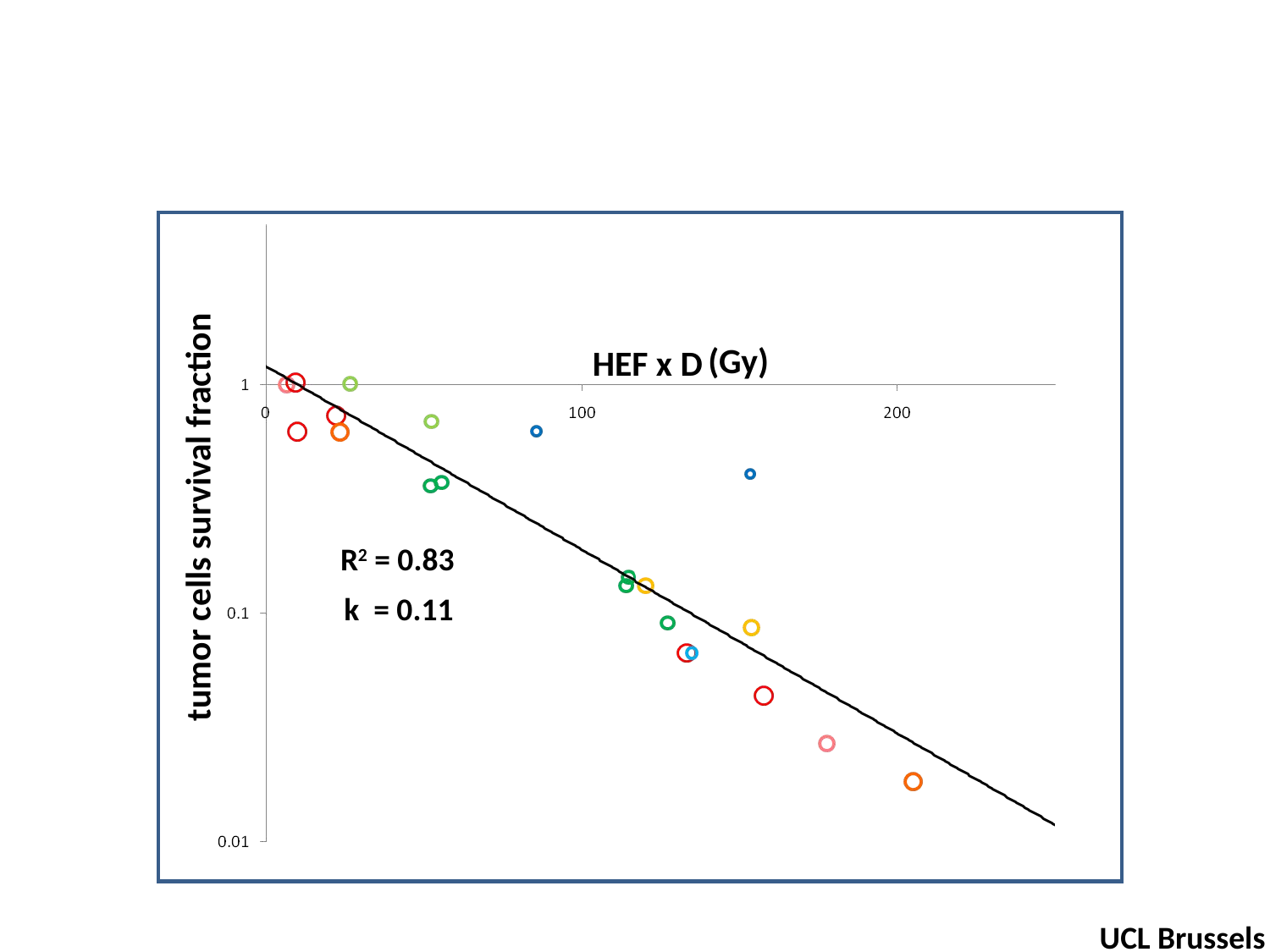

R2 = 0.83
(Gy)
tumor cells survival fraction
HEF x D
k = 0.11
UCL Brussels

## Slide 14
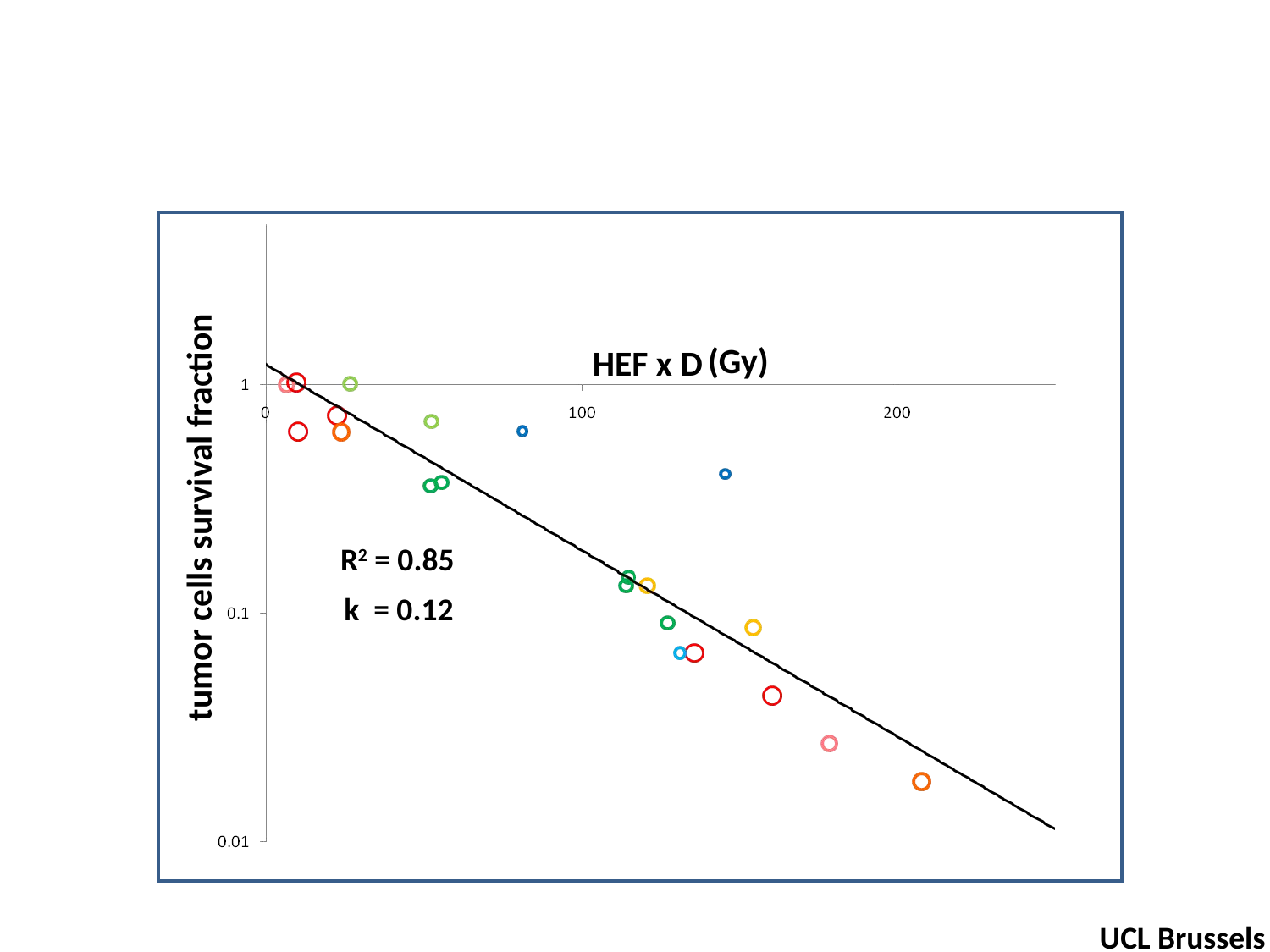

(Gy)
tumor cells survival fraction
R2 = 0.85
HEF x D
k = 0.12
UCL Brussels

## Slide 15
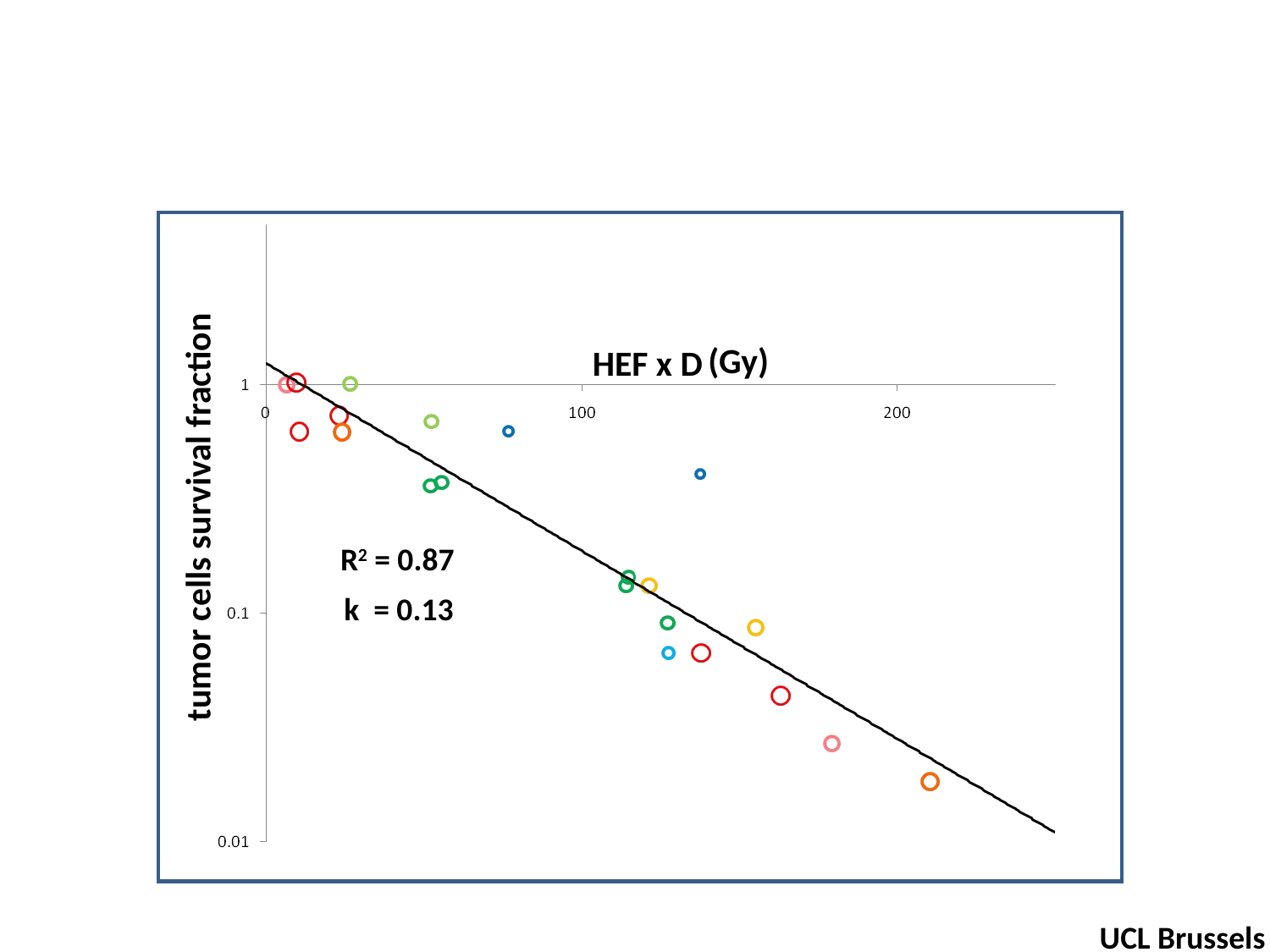

R2 = 0.87
(Gy)
tumor cells survival fraction
HEF x D
k = 0.13
UCL Brussels

## Slide 16
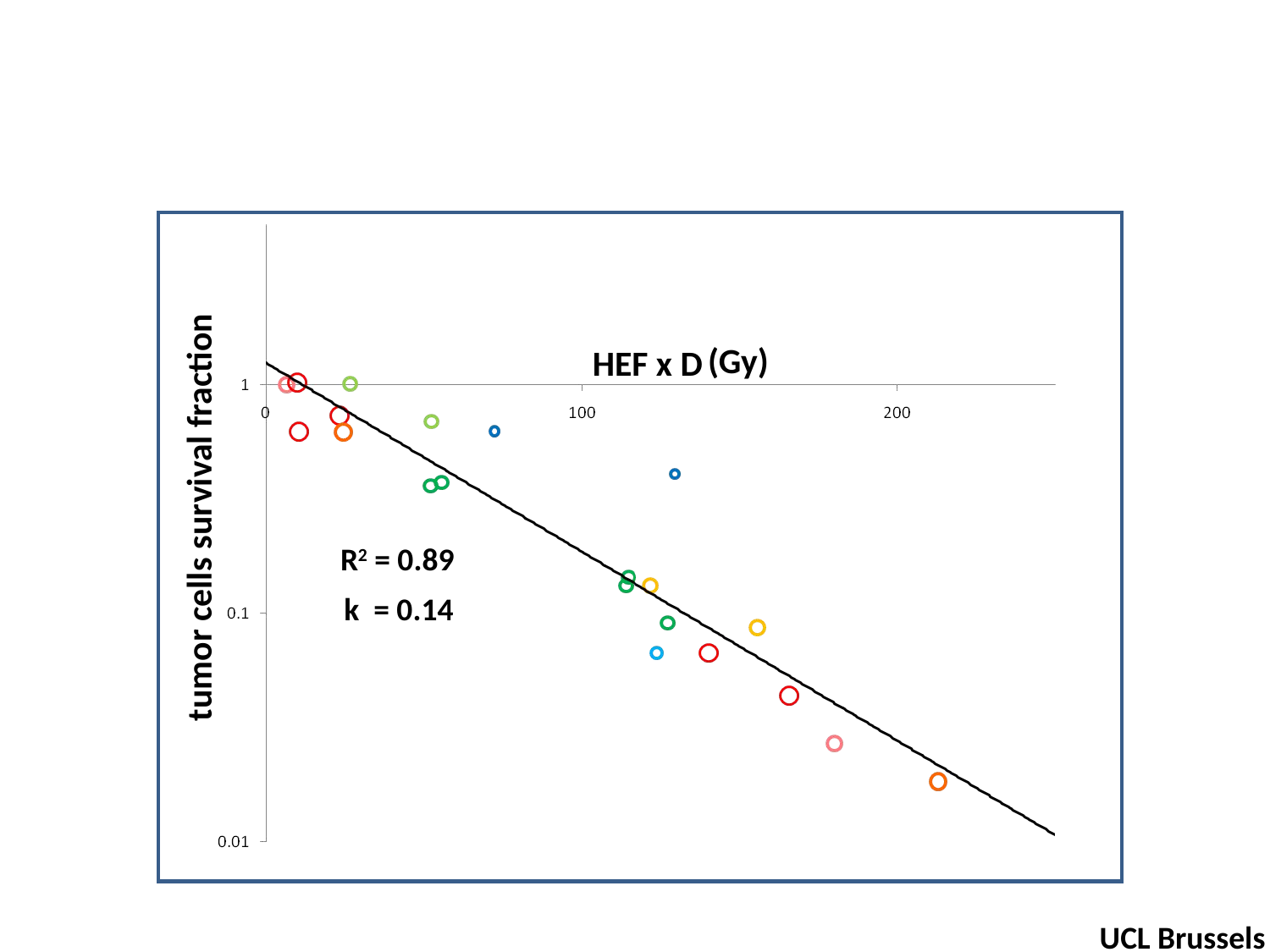

(Gy)
tumor cells survival fraction
R2 = 0.89
HEF x D
k = 0.14
UCL Brussels

## Slide 17
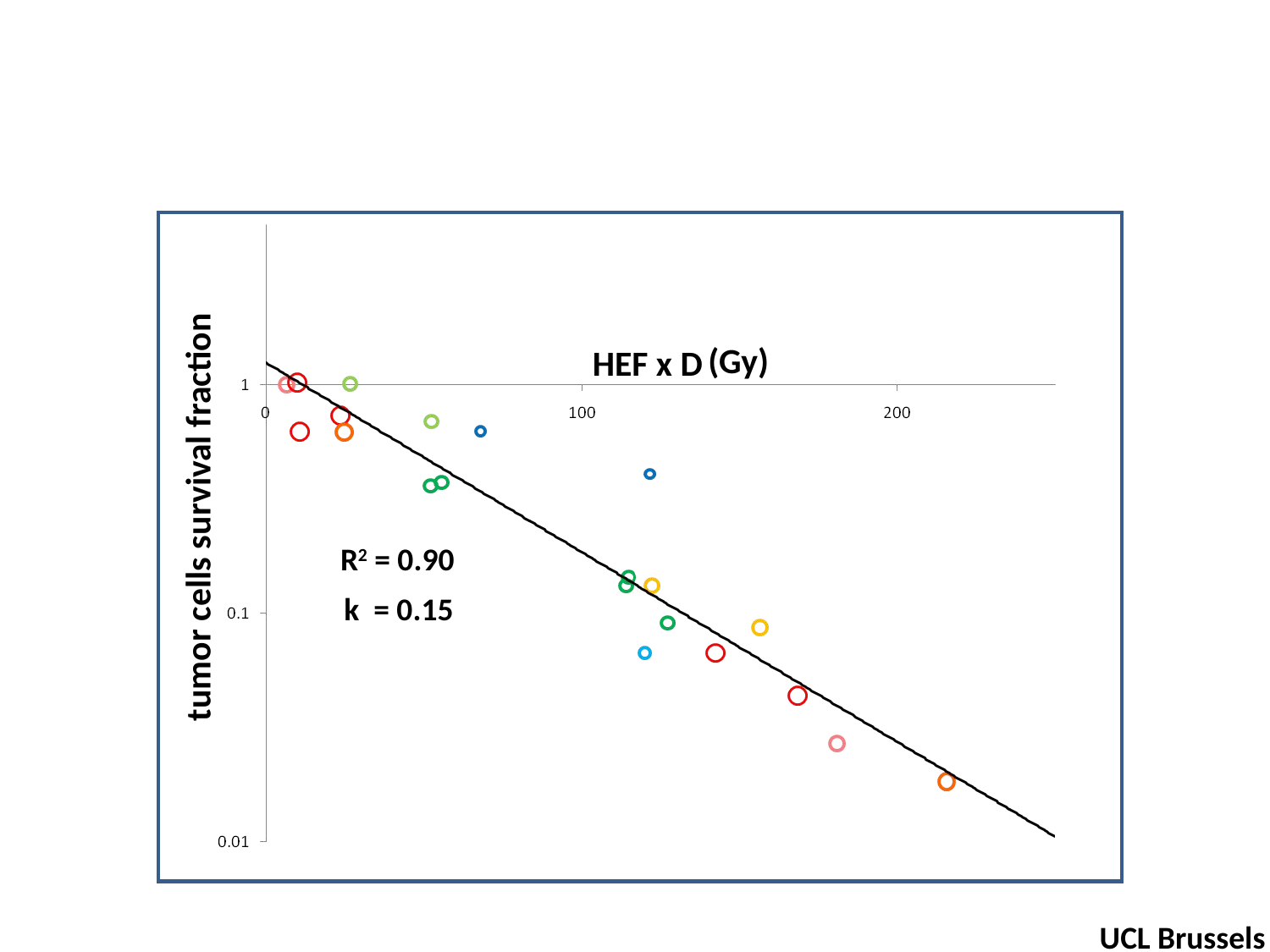

R2 = 0.90
(Gy)
tumor cells survival fraction
HEF x D
k = 0.15
UCL Brussels

## Slide 18
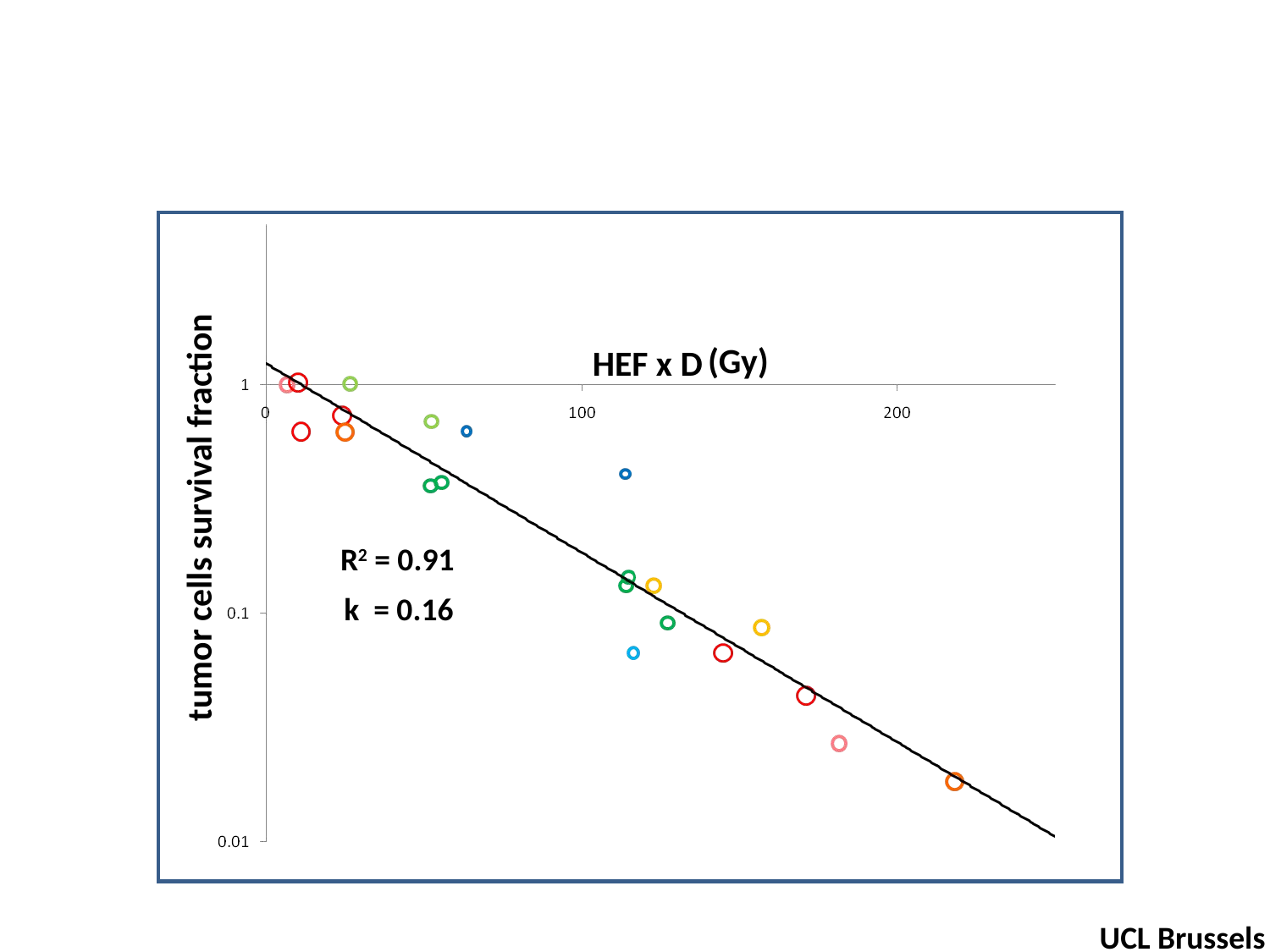

(Gy)
tumor cells survival fraction
R2 = 0.91
HEF x D
k = 0.16
UCL Brussels

## Slide 19
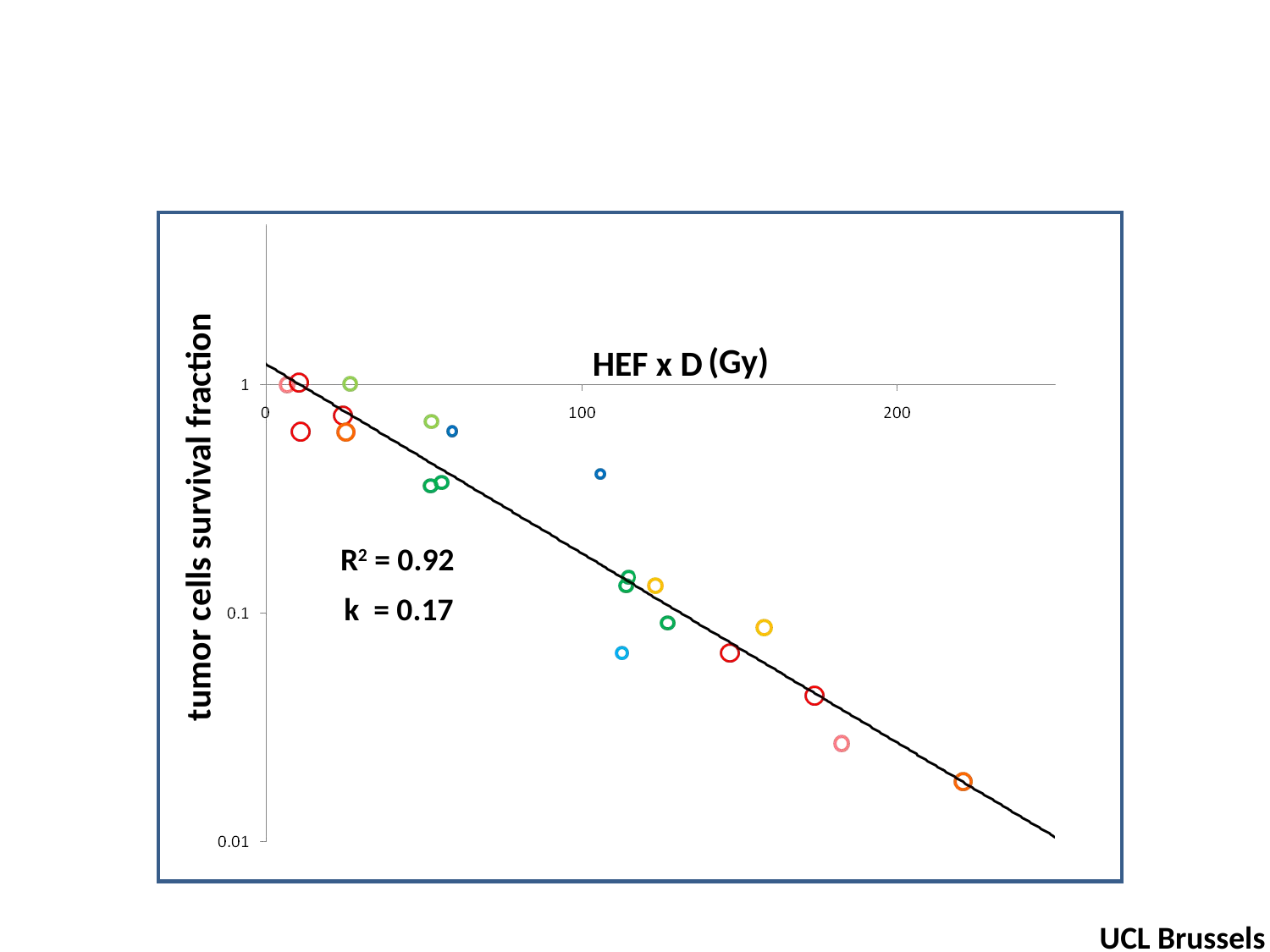

R2 = 0.92
(Gy)
tumor cells survival fraction
HEF x D
k = 0.17
UCL Brussels

## Slide 20
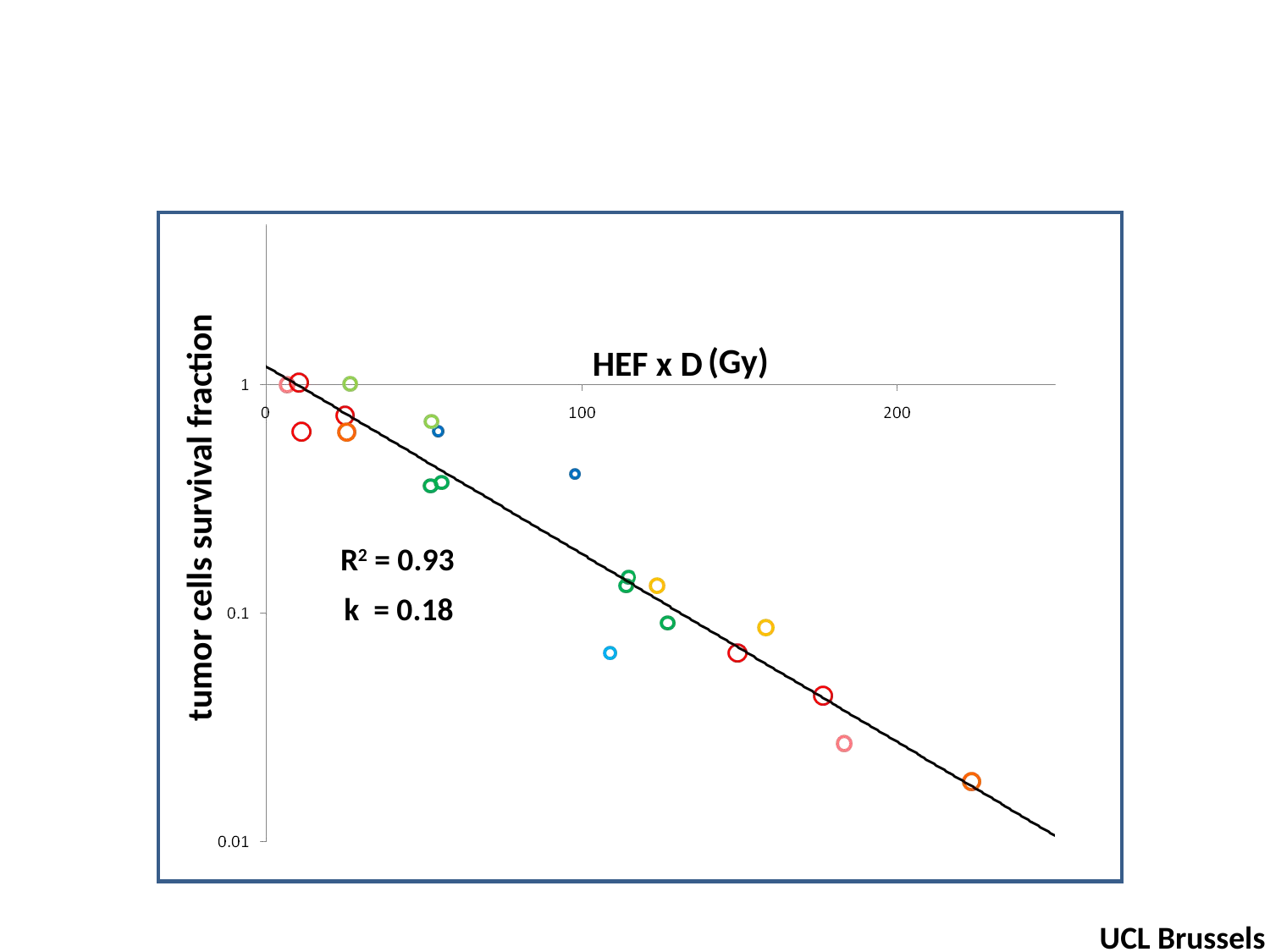

(Gy)
tumor cells survival fraction
R2 = 0.93
HEF x D
k = 0.18
UCL Brussels

## Slide 21
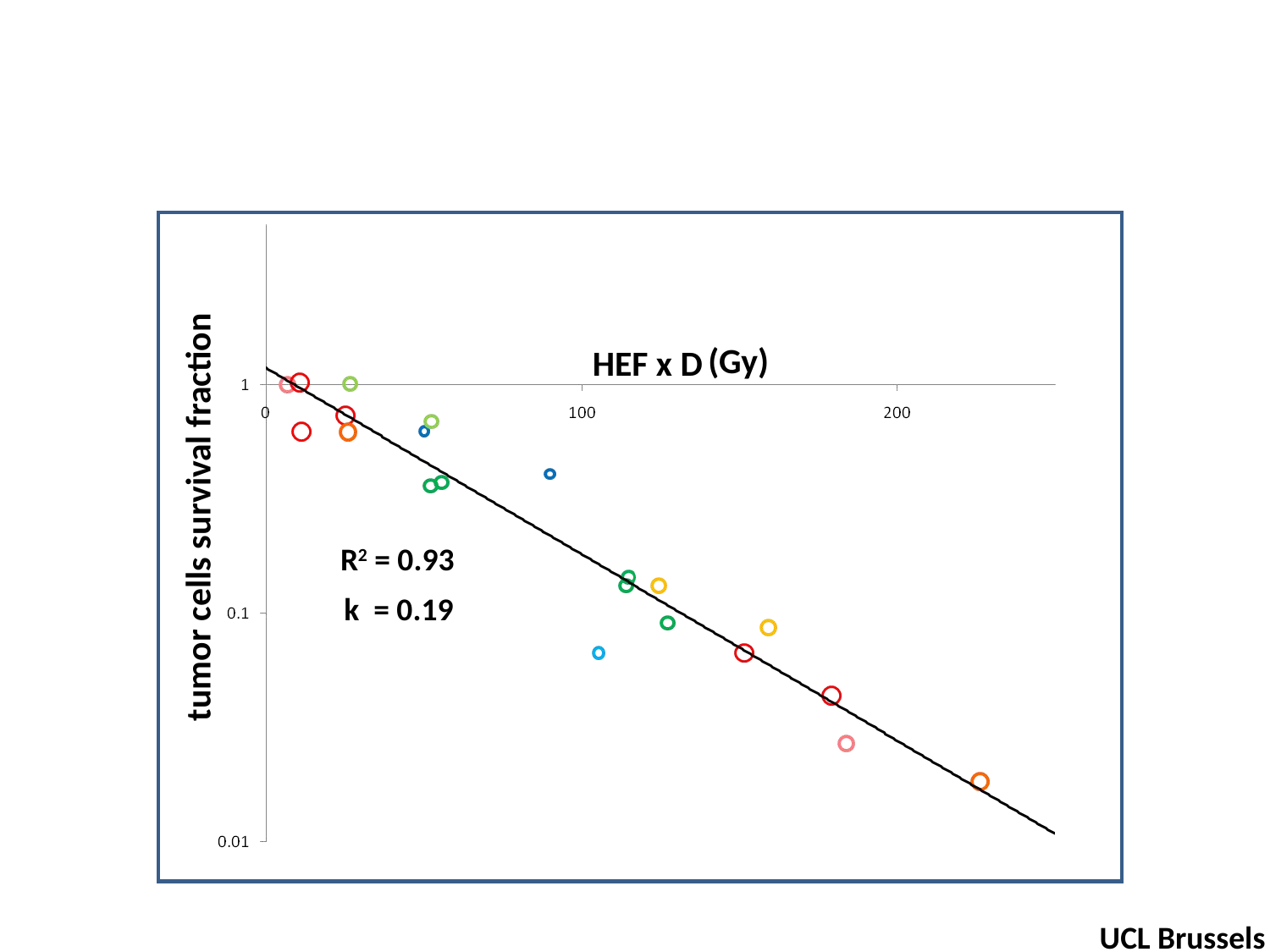

R2 = 0.93
(Gy)
tumor cells survival fraction
HEF x D
k = 0.19
UCL Brussels

## Slide 22
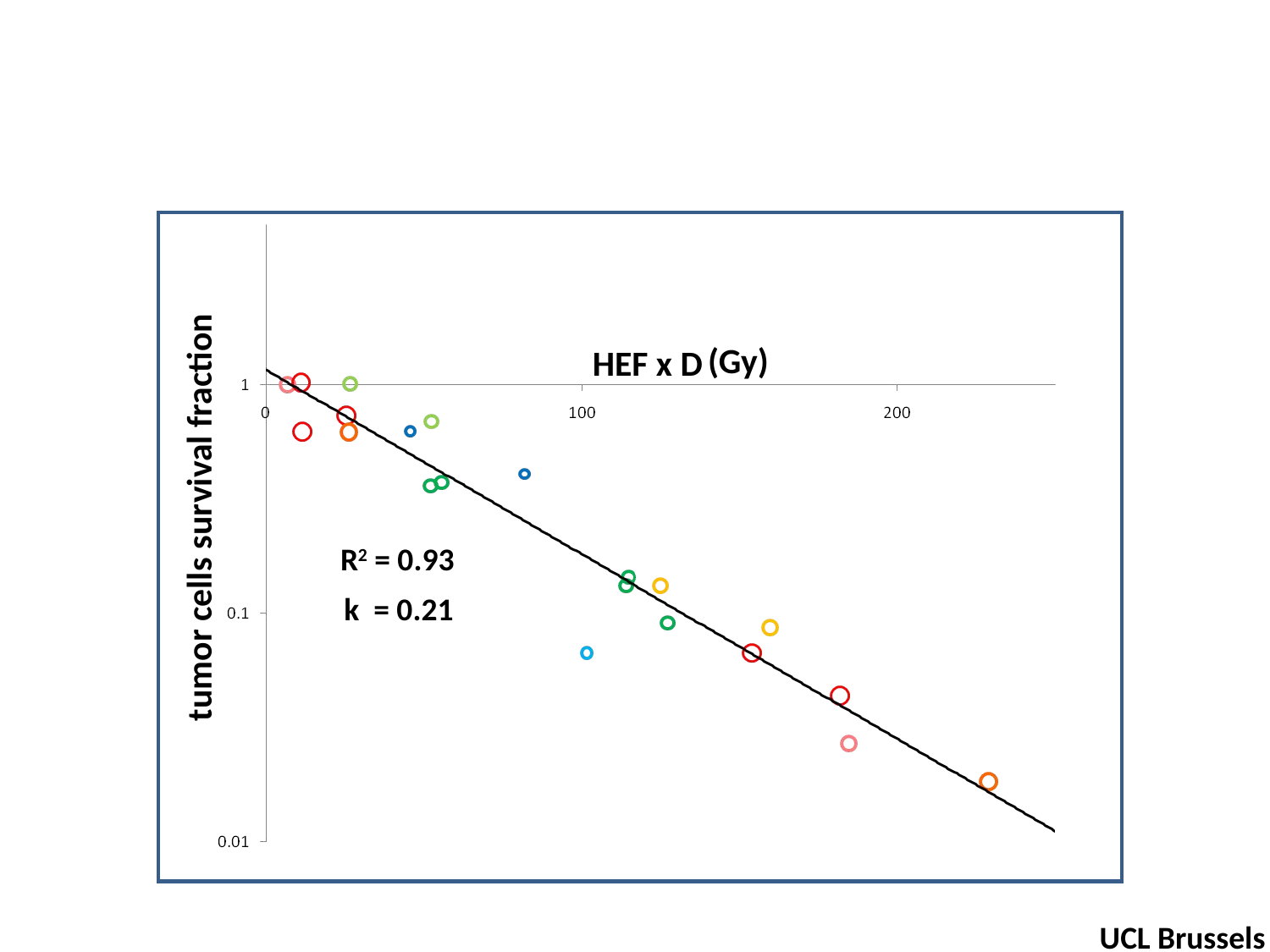

(Gy)
tumor cells survival fraction
R2 = 0.93
HEF x D
k = 0.21
UCL Brussels

## Slide 23
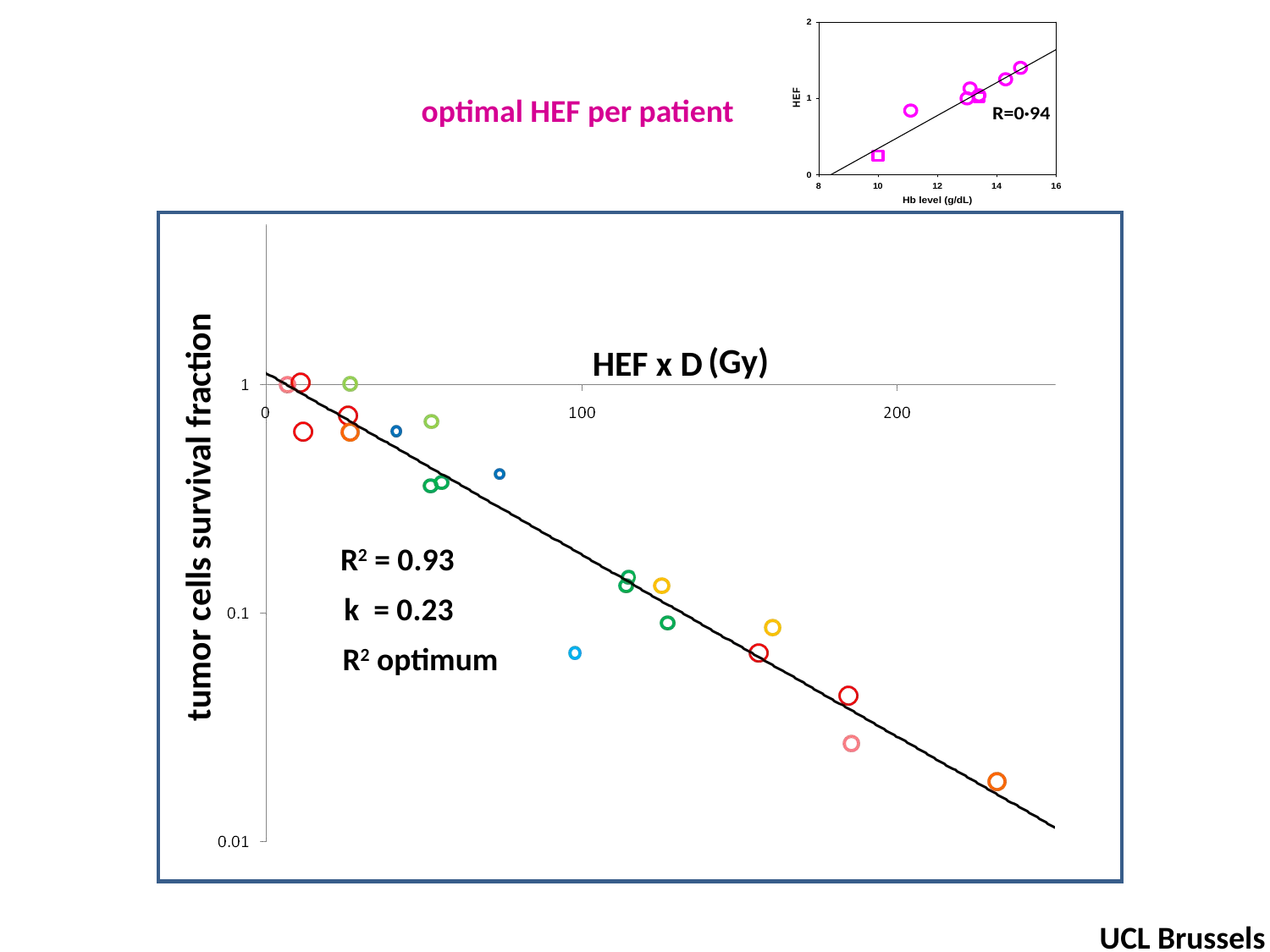

optimal HEF per patient
R2 = 0.93
(Gy)
tumor cells survival fraction
HEF x D
k = 0.23
R2 optimum
UCL Brussels
